# CD8^+^ T Cell-Based Molecular Classification With Heterogeneous Immunogenomic Landscapes and Clinical Significance of Clear Cell Renal Cell Carcinoma

**DOI:** 10.3389/fimmu.2021.745945

**Published:** 2021-12-14

**Authors:** Xiangkun Wu, Dongmei Jiang, Hongling Liu, Xiaofan Lu, Daojun Lv, Li Liang

**Affiliations:** ^1^ Department of Pathology, Nanfang Hospital and Basic Medical College, Southern Medical University, Guangzhou, China; ^2^ Department of Pathology & Guangdong Province Key Laboratory of Molecular Tumor Pathology, School of Basic Medical Sciences, Southern Medical University, Guangzhou, China; ^3^ Department of Pathology, The First Affiliated Hospital of Guangzhou Medical University, Guangzhou, China; ^4^ State Key Laboratory of Natural Medicines, Research Center of Biostatistics and Computational Pharmacy, China Pharmaceutical University, Nanjing, China; ^5^ Department of Urology, The Third Affiliated Hospital of Guangzhou Medical University, Guangzhou, China

**Keywords:** CD8^+^ T cells, immunogenomic analysis, immune checkpoint blockade therapy, clear cell renal cell carcinoma, unsupervised cluster analysis

## Abstract

The tumor microenvironment (TME) exerts a high impact on tumor biology and immunotherapy. The heterogeneous phenotypes and the clinical significance of CD8^+^ T cells in TME have not been fully elucidated. Here, a comprehensive immunogenomic analysis based on multi-omics data was performed to investigate the clinical significance and tumor heterogeneity between CD8^+^ T cell-related molecular clusters. We identified two distinct molecular clusters of ccRCC (C1 and C2) in TCGA and validated in E-MTAB-1980 cohorts. The C1 cluster was characterized by unfavorable prognosis, increased expression levels of CD8^+^ T cell exhaustion markers, high immune infiltration levels as well as more immune escape mechanisms. The C2 cluster was featured by favorable prognosis, elevated expression levels of CD8^+^ T cell effector markers, low load of copy number loss and low frequency of 9p21.3 deletion. Moreover, the effect of molecular classifications on Nivolumab therapeutic efficacy in the CheckMate 025 cohort was examined, and the C2 cluster exhibited a better prognosis. Taken together, we determine two CD8^+^ T cell-related molecular clusters in ccRCC, and provide new insights for evaluating the functions of CD8^+^ T cells. Our molecular classification is a potential strategy for prognostic prediction and immunotherapeutic guidance for ccRCC patients.

## Introduction

Globally, renal cell cancer (RCC) is the third most diagnosed genitourinary malignancy (1). Clear cell renal cell carcinoma (ccRCC) is the most common histological subtype of RCC (accounting for about 70% of cases) (2). Although nephrectomy is a good therapeutic option for controlling localized ccRCC, nearly 30% of patients subjected to nephrectomy experience recurrence or distant metastasis (3–5). Metastatic RCC (mRCC) is almost always fatal, with ten-year survival rates of less than 5% (6, 7). Targeted therapy for mRCC is less likely to significantly change the clinical outcomes (8). Invention of immune checkpoint blockade (ICB) therapy targeting the programmed cell-death protein 1 (PD-1)/programmed cell death 1 ligand 1 (PD-L1) axis and cytotoxic T-lymphocyte-associated protein 4 (CTLA-4) has revolutionized RCC treatment (9–12). However, studies have reported low ICB therapeutic response rates among mRCC patients (13). Therefore, there is an urgent need to identify novel markers for predicting the prognosis and ICB therapeutic efficacy for ccRCC.

Tumor intrinsic features (14, 15) and TME characteristics are involved in ICB therapeutic responses of solid tumors (16, 17). Studies have aimed at developing strategies to overcome the immunosuppressive TME, thereby, improving the efficacy of ICB therapies (18). Among tumor-infiltrating immune cells, T cells are highly correlated with the immunosuppressive characteristic of ccRCC (19). T cell exhaustion in the TME may be the main reason for the low ICB therapeutic response rates (10). Generally, T cell exhaustion means that the state of CD8^+^ T cells is converted from an anti-tumor status to an immune-functionally impaired status due to long-term persistence of tumor antigens and/or the suppressive TME (20). Therefore, conversion of exhausted T cells back to the activated state will have important clinical implications for ccRCC.

CD8^+^ T cells, one of the largest proportions of T cells in the TME, which are major drivers of anti-tumor immunity (21). Unlike many other solid tumors, high infiltration levels of CD8^+^ T cells has been reported to be associated with poor prognosis in ccRCC (22, 23). The CD8^+^ T cells in the ccRCC TME exhibited elevated expression levels of immune evasive biomarkers and enhanced immunosuppressive cell infiltrations (24, 25). However, the relationship between the degree of CD8^+^ T cell infiltration and the ICB therapeutic responses in ccRCC remains unclear, perhaps because CD8^+^ T cell infiltrated ccRCCs are enriched with 9p21.3 deletions and relatively depleted PBRM1 mutations (26). In addition, a subpopulation of CD8^+^ T cells has been closely associated with ICB therapeutic responses in ccRCC (27). These above findings indicated that CD8^+^ T cells present highly heterogeneous phenotypes in the TME of ccRCC. Thus, further evaluation of biomarkers and molecular clusters associated with CD8^+^ T cells is urgent needed, which would be helpful to identify new prognostic markers to inform ICB therapy for ccRCC.

In this study, we characterized CD8^+^ T cell-related molecular clusters to identify potential biological functions of CD8^+^ T cells in ccRCC. Firstly, WGCNA was performed to identify modules associated with CD8^+^ T cells in ccRCC. Subsequently, unsupervised cluster analysis was carried out to identify two distinct CD8^+^ T cell- related molecular clusters with different genomic alterations and clinical significance. Finally, a simple gene classier was constructed to inform on the prognosis and guide ICB therapy for ccRCC patients.

## Methods

### Dataset Acquisition and Preparation

The RNA-sequencing data (HTSeq-Counts) for ccRCC (n = 539) and normal (n = 72) tissue samples was downloaded from The Cancer Genome Atlas (TCGA) website (https://portal.gdc.cancer.gov/) while the corresponding clinical data of ccRCC samples was downloaded from the cBioportal website (https://www.cbioportal.org/datasets). Raw counts of RNA-sequencing data were transformed into transcripts per million (TPM) values, and were further log2- transformed (log2TPM) for subsequent analyses. Matrix files of gene expression profiles and clinical information of the E-MTAB-1980 cohort was downloaded from the ArrayExpress website (https://www.ebi.ac.uk/arrayexpress/experiments/E-MTAB-1980/).

Normalized transcriptomic and clinical data of CheckMate 025 (CM-025) cohorts of ccRCC patients treated with Nivolumab (anti-PD-1) therapy were obtained from the published article (26). The inclusion criteria for ccRCC samples were: i. Those with sequencing or array data; ii. Those with survival data; iii. Those with a follow-up of ≥ 30 days; and iv. Pathologically confirmed ccRCC cases. Samples from the CM-025 cohorts that did not have information regarding ICB therapeutic responses were excluded. Finally, 539 ccRCC and 72 normal tissue samples were used to identify differentially expressed genes (DEGs). A total of 509 ccRCC samples from TCGA, 101 from E-MTAB-1980 and 172 from CM-025 cohorts were enrolled in this study.

### Differential Analysis of Immune-Related Genes

A total of 7399 immune-related genes were obtained from the InnateDB website (https://www.innated b.com/redirect.do?go=resourcesGeneLists). Then, the “edgeR” R package (version 3.30.0) was used to identify DEGs between ccRCC (n = 539) and normal (n = 72) tissue samples in TCGA. The threshold for statistically significant DEGs was set at |log2-fold change (FC)| > 1 and false discovery rate (FDR) < 0.05. Then, immune-related genes were intersected with significant DEGs to obtain immune-related differentially expressed genes (IRDEGs).

### Acquisition and Survival Analysis of Immune and Non-Immune Cells in the TME

A total of 64 immune and non-immune cell types in the TME were downloaded from xCell (http://xcell.ucsf.edu/). xCell is an enrichment algorithm with 6573 gene signatures of 64 immune and non-immune cell types, including epithelial cells, hematopoietic progenitors, extracellular matrix cells, adaptive and innate immune cells. The xCell obtained the full cellular landscape of 64 human cell type from various sources and adopted an integrated approach combining the advantages of gene set enrichment and deconvolution approaches to identify cell types across data sources (28). Cox regression and Kaplan-Meier analyses were performed to identify prognostic immune and stroma cell types by “survival” R package. The “survminer” R package was used to determine the best cutoff.

### Weighted Correlation Network Analysis (WGCNA) of IRDEGs

WGCNA was performed using the “WGCNA” R package to identify IRDEGs associated with CD8^+^ T cells on the TCGA cohort. The expression profile of IRDEGs with log2TPM expression greater than 1 (n = 1339) derived from the above analysis was used as the input file for WGCNA. The WGCNA is a method for evaluating correlation patterns among genes across samples and for visualizing co-expression networks (29). Adjacency matrix was generated by Pearson’s correlation between all pair-wise genes. The soft threshold power of β = 6 was selected to achieve scale-free topology of the adjacency matrix. Then, the adjacency matrix was transformed into topological overlap matrix (TOM). According to TOM‐based dissimilarity measure with minimal module size as 30 and cut height as 0.25, IRDEGs with similar expression patterns were classified into the same gene module by average linkage hierarchical clustering. Then, we evaluated the correlation between module eigengenes (MEs) and clinical traits to identify clinically significant modules.

### Unsupervised Cluster Analysis

Kaplan-Meier and univariate Cox regression analyses were performed using the “survival” R package to select genes from the brown module, and genes with p < 0.01 were selected for unsupervised cluster analysis. Then, we performed the consensus non-negative matrix factorization (CNMF) algorithm based on above genes and selected default parameters for molecular classification of the TCGA cohort using the “CancerSubtype” R package (30). The CNMF algorithm is a classical dimension reduction method that extracts essential data from high-dimensional data. Since the CNMF function of the “CancerSubtype” R package is designed for multi-omics data analysis, we randomly divided gene expression profiles into two datasets, as two different omics. Silhouette coefficient, which ranged from -1 to 1, was used to estimate the result of the cluster. Silhouette coefficients near 1 indicate that the sample is distinguished from neighboring clusters and was used to determine the best number of clusters (31). The same classification procedure was performed for the E-MTAB-1980 cohort to validate molecular classification.

### Prognostic Analysis of Molecular Clusters

Cox regression analysis was performed to assess the prognostic significance of molecular clusters in the TCGA cohort. Moreover, the independence of molecular classification based on clinical characteristics (T stage, M stage, Stage and Grade) was also evaluated. Then, time-dependent receiver operating characteristic (ROC) analysis was compared with area under the curve (AUC), concordance index (C-index) and decision curve analysis (DCA) between molecular clusters and ClearCode34 (32). Brooks et al. developed a model based on 34 gene expressions to classify ccRCC patients and to predict patient survival outcomes (32). The “compare” function in “timeROC” R package and the “cindex.comp” function in “survcomp” R package were used to statistically test the difference in AUC and C-index between different signatures, respectively. Decision curve analysis (DCA) was performed to evaluate the net benefits derived from the use of molecular classification and ccA/ccB subtypes. Subsequently, the associations between molecular clusters, clinical characteristics and ClearCode34 were evaluated. The sankey diagram was used to visualize relationships among molecular clusters, stage, grade, survival status and ClearCode34.

### Somatic Mutations and Copy-Number Alterations (CNAs) Data

Data for somatic mutations and CNAs were downloaded from the cBioportal website (https://www.cbioportal.org/datasets). Genes with mutation rates greater than 2% were included in this study. Copy-number alterations data were analyzed using the GISTIC 2.0 online version (https://cloud.genepattern.org/gp/pages/index.jsf). Threshold copy numbers at alteration peaks (kirc.all_lesions.conf_90.txt file) were obtained by GISTIC analysis. CNAs with occurrence rates greater than 2% were also included in this study. CNAs were divided into focal and arm levels by GISTIC according to the length of genome mutation fragments. Focal CNAs were defined as shorter than the chromosome arm and arm CNAs were defined as chromosome-arm length or longer (33). The load of copy number loss or gain was defined as the total number of CNAs at focal and arm levels.

### Acquisition of Immunogenomic Signatures

Immune-related features, including tumor mutation burden (TMB), neoantigen load, homologous recombination defects (HRD), CTA scores and intratumor heterogeneity (ITH) were also retrieved (34). Immune scores for each TCGA sample were downloaded from the ESTIMATE website (https://bioinformatics.mdanderson.org/estimate/index.html). A total of 178 immunomodulators and chemokines were obtained from the TISIDB website (http://cis.hku.hk/TISIDB/index.php), while 44 immunomodulators and chemokines without corresponding expression profiles were excluded.

### Single Sample Gene Set Enrichment Analysis (ssGSEA)

The immune suppression score signature (CD274, IDO1, FASLG, CTLA4, PDCD1, LAG3, HAVCR2, PDCD1LG2, IL10, TGFB1, PTGS2) (35, 36) and the signatures of 10 oncogenic pathways were obtained from a previously published study (37). ssGSEA algorithm of “GSVA” R package would rank-normalized gene expression values for each ccRCC sample, and a normalized enrichment scores (NES) of each ccRCC sample was generated using the Empirical Cumulative Distribution Functions of the genes and the remaining genes in each signature (38, 39). The NES represented the degree of absolute enrichment of a gene signature in each ccRCC sample, which can reflect the activity of a signature or a pathway. Subsequently, NES were compared among different clusters.

### Functional Enrichment Analysis

Annotated gene sets c2.cp.kegg.v7.2.entrez.gmt, c5.go.v7.2.entrez.gmt, c6.all.v7.2.entrez.gmt, c7.all.v7.2.entrez.gmt, c8.all.v7.2.entrez.gmt and h.all.v7.4.entrez.gmt were downloaded from the GSEA website (http://www.gsea-msigdb.org/gsea/downloads.jsp). Comprehensive functional enrichment analyses, including GO enrichment analysis, KEGG enrichment analysis, oncogenic signature, immune signature, cell type signature and “hallmark” signature were performed using “clusterprofiler” R package. Hallmark pools specific well-defined biological states or processes and demonstrates coherent expression, which was extensively utilized in medical studies (40–44). FDR < 0.05 was considered statistically significant.

### Gene Set Variation Analysis (GSVA)

GSVA, a non-parametric estimation method, is commonly used for variation analyses of biological pathways between different molecular clusters. By using the “c2.cp.kegg.v6.2.symbols” gene set as the reference gene set, we performed GSVA to identify differences in KEGG pathways among different clusters and selected default parameters using the “GSVA” R package. The FDR < 0.05 and t value > 2 were set as the cut‐off criteria.

### Construction and Validation of the Gene Classifier

Based on the top 30 up-regulated DEGs in each cluster, we constructed a simple gene classifier to predict the molecular clusters using nearest template prediction (NTP) function of “CMScaller” R package. The NTP algorithm provides a convenient method for cluster prediction in a testing dataset using a list of signature genes of each cluster, which can be used to single-patient, multi-cluster and cross-platform predictions (45). This method has been successfully applied to clinical classification and prognosis prediction based on gene expression (46, 47). Therefore, this method was suitable for this study to establish a convenient gene classifier for clinical application. Then, the gene classifier was validated in E-MTAB-1980 and CM-025 cohorts and was compared with previous molecular classifications based on the CNMF algorithm. Sankey diagram was used to visualize the relationships between molecular classification and the gene classifier.

### Statistical Analyses

R software (https://www.r-project.org/) was used for all computational and statistical analyses. Cluster quality measure- “in-group proportion (IGP)” analysis was performed to evaluate reproducibility of the molecular clusters, and IGP close to 100% meant credible reproducibility of the clusters (48). Expression data of different datasets were normalized by Z-scores prior to IGP analysis. Determination of statistically significant DEGs between two TCGA clusters using the “limma” package were defined as |log2FC| > 1 and FDR < 0.05. The Hazard Ratio (HR) and 95% confidence interval (CI) were generated by Kaplan-Meier and Cox regression analyses. Statistical significance of the comparisons between two groups for continuous variables and categorical variables was estimated by the Student’s T test or Mann-Whitney U test and Chi-square test or Fisher’s exact tests, respectively. Correlations between variables were assessed by Spearman correlation analysis. Two-tailed p ≤ 0.05 was considered statistically significant.

## Results

### Identification of IRDEGs in ccRCC

The flowchart of this study was shown in **Figure S1A**. We compared the RNA expression levels between ccRCC (n = 539) and normal tissues (n = 72) in the TCGA cohort (|log2FC| > 1, FDR < 0.05), and obtained a total of 5640 DEGs (3726 upregulated and 1914 downregulated) (**Supplement Table 1**). The volcano plot showed the distribution of all DEGs in TCGA (**Figure S1B**). We obtained a total of 1399 IRDEGs with the expression greater than log2TPM 1 through the intersection of DEGs and InnateDB immune-related genes (**Figure S1C** and **Supplement Table 2**).

### Survival Analysis of 64 Immune and Non-Immune Cell Types

**Figure 1:**
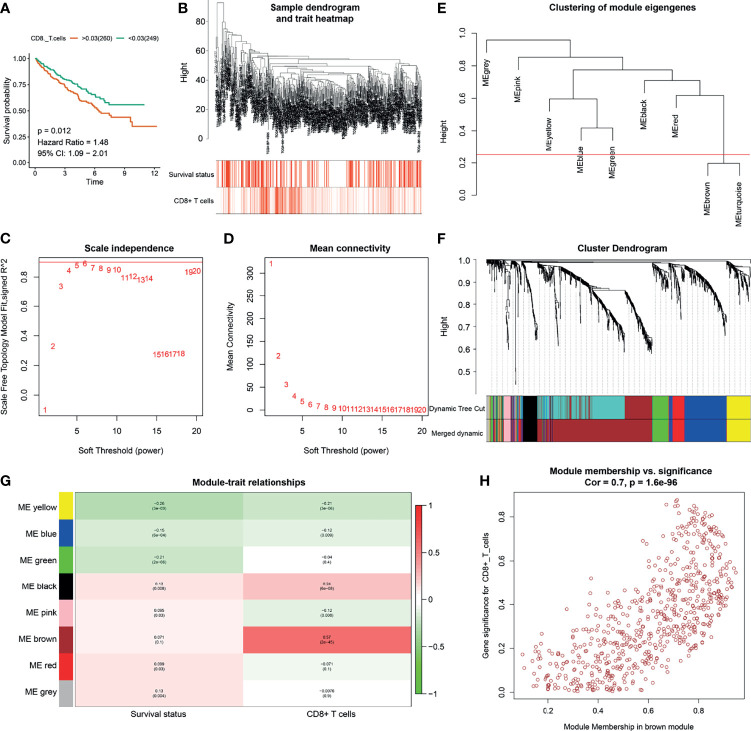
Identification of CD8^+^ T cell related genes. **(A)** Kaplan-Meier analysis of CD8^+^ T cells. **(B)** Clustering dendrograms of immune-related differentially expressed genes (IRDEGs). Color intensity varies positively with abundance of CD8^+^ T cells. In terms of survival status, red means dead and white indicates live. **(C, D)** Analysis of scale-free fit index **(C)** and mean connectivity **(D)** for various soft-thresholding powers. **(E)** Clustering of module eigengenes. The red line shows cut height (0.25). **(F)** Dendrogram of robust IRDEGs clustered based on a dissimilarity measure (1-TOM). **(G)** Heatmap of the correlation between module eigengenes and clinical traits of ccRCC. Each cell contains p-value and the correlation coefficient. **(H)** Scatter plot of module eigengenes related to abundance of CD8^+^ T cells in the brown module.

Kaplan-Meier and Cox regression analyses showed that out of 64 immune and non-immune cells, 21 cell types were associated with overall survival of ccRCC. (**Figure S2** and **Supplement Table 3**). Multiple lymphoids were associated with poor prognosis, including CD8^+^ T cells, B-cells, Th1 cells, effector memory CD8^+^ T cell (CD8^+^ Tem), natural killer T cells (NKT), Plasma cells and Th2 cells (**Figure 1A** and **Figure S2**). In myeloid cells, eosinophils and conventional dendritic cells (cDC) were associated with good prognosis whereas basophils, mesangial cells, M1 Macrophages and monocytes were associated with poor prognosis. High abundance of CD8^+^ T cells was associated with worse prognosis, which consistent with previous studies (22, 23).

### Weighted Gene Correlation Network Analysis of IRDEGs

The abundance of CD8^+^ T cells was extracted from the xCell website (http://xcell.ucsf.edu/). To identify key modules that were significantly correlated with the abundance of CD8^+^ T cells, we performed WGCNA on the TCGA-ccRCC dataset after incorporating the 1399 IRDEGs derived from the above analysis (**Figure 1**). Clustering dendrograms of IRDEGs was shown in **Figure 1B** and color intensity varies were positively with abundance of CD8^+^ T cells. In terms of survival status, red means dead and white indicates live **(** **Figure 1B**). Analysis of scale-free fit index and mean connectivity for various soft-thresholding powers was shown in **Figures 1C, D**, respectively. By setting the cut height = 0.25, the brown and turquoise module eigengenes were combined (**Figures 1E, F** **)**. **Figure 1F** also show the dendrogram of robust IRDEGs clustered based on a dissimilarity measure. By setting the cut height = 0.25 and β = 6 (scale-free R2 = 0.85), 1399 IRDEGs were divided into eight independent co-expression modules (**Figure 1G**). As shown in the relative diagram of module-trait relationship, the brown module including 648 IRDEGs was most significantly correlated with the abundance of CD8^+^ T cells (**Figures 1G, H** and **Supplement Table 4**).

### Heterogenous Phenotypes of CD8^+^ T Cells

**Figure 2:**
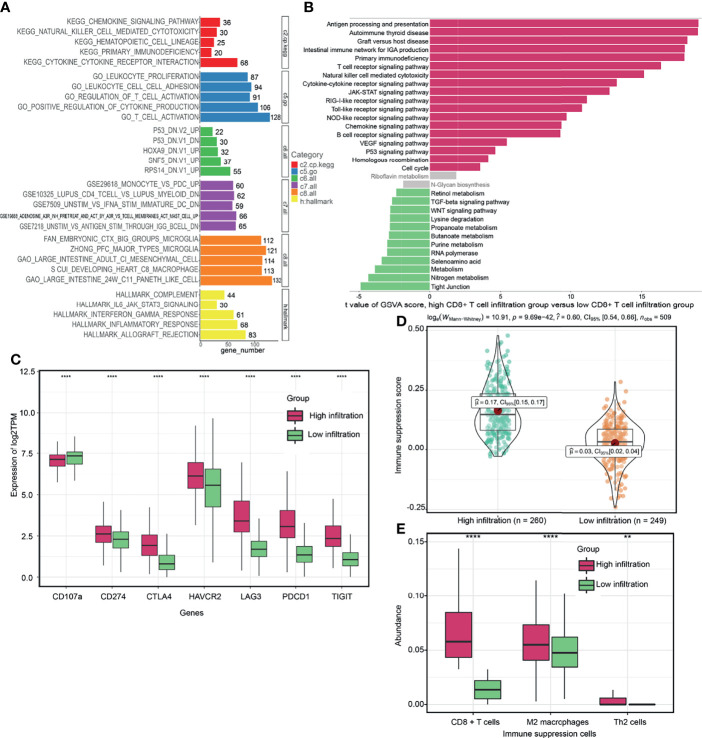
Potential biological functions of CD8^+^ T cells. **(A)** Comprehensive functional enrichment analysis of CD8^+^ T cell related genes. **(B)** Gene set variation analysis (GSVA) between high CD8^+^ T cell- infiltration and low CD8^+^ T cell- infiltration groups. **(C, D)** Differences of immune-related signatures between high CD8^+^ T cell- infiltration and low CD8^+^ T cell- infiltration groups. **(E)** The abundance of each immunosuppressive cell in high CD8^+^ T cell- infiltration and low CD8^+^ T cell- infiltration groups. (****P < 0.0001; **P < 0.01).

To evaluate the potential biological mechanisms of CD8^+^ T cells, we performed comprehensive functional enrichment analysis of CD8^+^ T cell related genes using the “cluster profiler” R package. **Figure 2A** and **Supplement Table 5** showed that immune-related KEGG pathways, such as chemokine signaling pathway, natural killer cell mediated cytotoxicity, primary immunodeficiency, cytokine-cytokine receptor interaction and hematopoietic cell lineage were enriched. The GO term, including leukocyte proliferation, leukocyte cell-cell adhesion, regulation of T cell activation, positive regulation of cytokine production and T cell activation were mostly associated with CD8^+^ T cell related genes. “Hallmark” gene signatures analysis revealed that CD8^+^ T cells could be involved in multiple biological states or processes, such as complement, IL6 Janus kinase (JAK)-signal transducer and activator of transcription (STAT) signaling (signaling, interferon Gamma (INFγ) responses, inflammatory responses, and allograft rejection. Furthermore, based on the cutoff identified by the “survminer” R package, we divided the TCGA cohorts into two groups, high CD8^+^ T cell- infiltration and low CD8^+^ T cell- infiltration groups. The results from GSVA analysis revealed that the low CD8^+^ T cell- infiltration group was enriched with more metabolism-related processes (e.g., tyrosine metabolism, selenoamino acid metabolism, purine metabolism, butanoate metabolism and propanoate metabolism et al), as well as WNT signaling and transforming growth factor (TGF)-β signaling pathways (**Figure 2B** and **Supplement Table 6** **)**. High CD8^+^ T cell- infiltration group were related to many immune-related biological pathways, such as intestinal immune network for IGA production, antigen processing and presentation, T cell receptor signaling pathway, natural killer cell mediated cytotoxicity and cytokine receptor interactions et al, but more related to immunosuppressive pathways, such as primary immunodeficiency, vascular endothelial growth factor (VEGF) signaling, hedgehog signaling, p53 signaling, JAK-STAT signaling, and Toll like receptor signaling (**Figure 2B**). Meanwhile, the abundance of CD8^+^ T cells was positively correlated with immune suppression scores and expression of critical immune checkpoint genes (CTLA4, TIGIT, PDCD1, LAG3 and CD274) (**Figures S3B–F**). However, it was negatively correlated with the expression of CD107a (T cell degranulation related factors) (**Figure S3G**). The TMB or neoantigen load of ccRCC was not correlated with the abundance of CD8^+^ T cells (**Figures S3H, I** **)**. The high CD8^+^ T cell- infiltration group exhibited elevated expression levels of critical immune checkpoint genes (**Figure 2C**). Previous studies reported that the TMB, neoantigen load and immune checkpoint gene expression might not be better indicators for ICB therapeutic efficacy in ccRCC, when compared to other solid tumor types (10, 49). Various subpopulations of CD8^+^ T cells were associated with ICB therapeutic responses in ccRCC (27, 50, 51). However, the above findings suggested that the CD8^+^ T cells and immune checkpoint gene expression, but not mutation or neoantigen loads, might be ideal predictors for ICB therapy. In addition, the high CD8^+^ T cell- infiltration group exhibited lower expression levels of CD107a and elevated immune suppression scores and abundance of immunosuppressive cells (M2 macrophages and Th2 cells) (**Figures 2C–E**), which may explain why ccRCC tumors progress despite high T cell infiltration. Overall, through functional enrichment analysis of CD8^+^ T cell- related genes, new insights can be provided for evaluating the heterogenous phenotypes of CD8^+^ T cells.

### Construction and Clinical Significance Analysis of Molecular Classification

**Figure 3:**
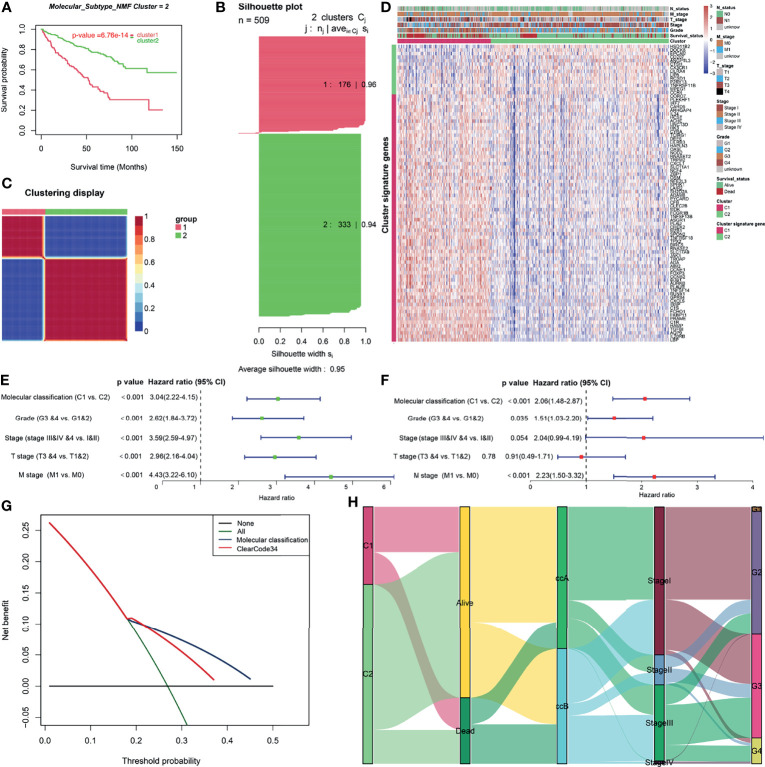
Construction and clinical significance analysis of molecular classification in TCGA cohort. **(A)** Kaplan-Meier analysis showed the Cluster1 patients had significantly poorer prognosis than Cluster2 patients. **(B)** Silhouette coefficients near 1 indicate that the sample is distinguished from neighboring clusters and determine that the best number of clusters was two. **(C)** Differential expression tested the expression difference between two clusters. **(D)** Heatmap of the specific cluster-associated genes. Forrest plot of univariate **(E)** and multivariate **(F)** Cox regression analysis in the TCGA cohort. **(G)** DCA for 5-year OS prediction shows that the molecular cluster (Cluster) has higher net benefit across 0% to 50% threshold probabilities than ClearCode34 classification (Classification). **(H)** A Sankey plot was used to reveal the correlation between molecular cluster, ClearCode34 classification, and clinical characteristics. (DCA, decision curve analysis; TCGA, The Cancer Genome Atlas; OS, overall survival).

To retrieve key genes that were significantly associated with OS, univariate cox regression analysis was used to screen out the prognosis-related brown genes (p_Cox_ < 0.01). Then, the prognosis-related brown genes obtained from univariate cox regression analysis were further screen by Kaplan-Meier analysis (p_KM_ < 0.01, **Supplement Table 7**). Using the 84-gene panel, we performed molecular classification using the CNMF algorithm to characterize two distinct ccRCC molecular clusters; C1 cluster (N= 176 samples) and C2 cluster (N= 333 samples) (**Figure 3**). The C2 cluster exhibited a better OS than the C1 cluster (Log rank p < 0.001, **Figure 3A**). In addition, we attempted to divide ccRCC patients of TCGA cohort into 2, 3, 4 and 5 clusters, and found that the silhouette coefficients near 1were the largest when they were divided into two clusters (**Figure 3B** and **Figures S4A–C**). Therefore, we choose two clusters as the best clusters. Differential expression tested the expression difference between two clusters (**Figure 3C**). The C1 cluster was correlated a higher histological grade, T stage, N status, M stage and stage as shown in the heatmap (**Figure 3D** and **Table 1**). The heatmap showed that 84 prognostic genes were correlated with the abundance of CD8^+^ T cells in the TCGA cohort (**Figure S4D**).

**Table 1:** Clinicopathological characteristics of ccRCC patients in the TCGA cohort.

Characteristics	All Patients	Cluster1	Cluster2	p-value
Patients, no. (%)	509 (100)	176 (34.6)	333 (65.4)	
T stage, no. (%)				<0.001
T1	261 (51.3)	58 (33.0)	203 (61.0)	
T2	68 (13.3)	23 (13.1)	45 (13.5)	
T3	170 (33.4)	87 (49.4)	83 (24.9)	
T4	10 (2.0)	8 (4.5)	2 (0.6)	
N status, no. (%)				0.005
N0	227 (44.6)	78 (44.3)	149 (44.7)	
N1	15 (2.9)	11 (6.3)	4 (1.2)	
Unknown	267 (52.5)	87 (49.4)	180 (54.1)	
M stage, no. (%)				<0.001
M0	406 (79.8)	125 (71.0)	281 (84.4)	
M1	76 (14.9)	46 (26.1)	30 (9.0)	
Unknown	27 (5.3)	5 (2.9)	22 (6.6)	
Stage, no. (%)				<0.001
Stage I	255 (50.1)	54 (30.7)	201 (60.4)	
Stage II	56 (11.0)	19 (10.8)	37 (11.1)	
Stage III	115 (22.6)	54 (30.7)	61 (18.3)	
Stage IV	83 (16.3)	49 (27.8)	34 (10.2)	
Grade, no. (%)				<0.001
1	12 (2.4)	1 (0.6)	11 (3.3)	
2	217 (42.6)	45 (25.6)	172 (51.7)	
3	200 (39.3)	72 (40.9)	128 (38.4)	
4	72 (14.1)	56 (31.8)	16 (4.8)	
Unknown	8 (1.6)	2 (1.1)	6 (1.8)	
Survival status, no. (%)				<0.001
Alive	343 (67.4)	83 (47.2)	260 (78.1)	
Dead	166 (32.6)	93 (52.8)	73 (21.9)	
Survival time				<0.001
Median months (range)	45.8 (1-149.0)	38.8 (1-133.6)	49.5 (37.5-149.0)	

To evaluate the independence of molecular classification from clinical characteristics, a total of 475 ccRCC patients **(** **Supplement Table 8**) with complete clinical data, including T stage, M stage, stage, and grade, were selected for Cox regression analysis. After the adjustment of clinical characteristics (T stage, M stage, Stage and Grade), molecular classification was found to be an independent prognostic factor for OS outcomes of ccRCC (**Figures 3E, F** **)**. The 5-year AUC and 5-year C-index of molecular classification were somewhat higher than those of ClearCode34 (5-year AUC: 0.65 vs. 0.64; 5-year C-index: 0.64 vs. 0.61), but no statistical significance (p>0.05; **Figures S4E, F** **)**. Moreover, the DCA for 5-year OS prediction showed that the net benefit across 0% to 50% threshold probabilities of molecular classification was higher than that of ClearCode34 (**Figure 3G** **)**. Sankey diagram was used to visualize the relationships among molecular clusters, ClearCode34 subtypes and clinical characteristics (**Figure 3H**). Overall, we successfully construct two CD8^+^ T cell-based molecular classifications to predict the OS for ccRCC. The molecular classification in this study was of greater significance for the prediction of clinical prognosis.

### Validation of Molecular Classification in the E-MTAB-1980 Cohort

The same classification pipeline was performed on the E-MTAB-1980 cohort to validate the stability of molecular classification, and the result of Kaplan-Meier analysis was consistent with that of the TCGA cohort (**Figures S5A–C**). Furthermore, we attempted to divide ccRCC patients of E-MTAB-1980 cohort into 2, 3, 4 and 5 clusters, and found that the silhouette coefficients of two clusters were the largest. Therefore, we choose two clusters as the best clusters. The IGP values were 89.5% and 98.4% for C1 and C2 clusters, respectively, indicating that the two molecular clusters have a high degree of reproducibility in E-MTAB-1980 cohort.

### Differences in Biological Mechanisms Between Two Molecular Clusters

To investigate differences in biological mechanisms between molecular clusters, GSVA was performed in the TCGA cohort. The results showed that both of two molecular clusters were notably enriched in metabolism related pathways (**Figure S6A** and **Supplement Table 9**). Strikingly, gene sets related to primary immunodeficiency, nucleotide metabolism, JAK-STAT signaling, Toll like receptor signaling, vascular endothelial growth factor (VEGF) signaling, hedgehog signaling, p53 signaling and cell cycle/apoptosis related pathways were significantly elevated in the C1 cluster. In terms of the C2 cluster, it was dramatically enriched in amino acid metabolism (e.g tyrosine and glutamate metabolism), mammalian target of rapamycin (mTOR) signaling, transforming growth factor (TGF)-β signaling, insulin signaling, peroxisome proliferators-activated receptors (PPARs) signaling and tight junction pathways. It has been reported that these pathways might modify the cancer-related immune microenvironment. For example, previous studies have revealed that VEGF had direct or indirect effects on components of the immune system, including suppressing DC maturation and CD8^+^ T cell proliferation and regulating ICAM1 to suppress NK cell and T cell trafficking, resulting in immunosuppressive outcomes (52). Therefore, it could be deduced that the C1 cluster might develop cancer immune escape through multiple immunosuppressive pathways.

### Existing of High Immune Infiltration but Extrinsic Immune Escape Mechanisms in C1 Cluster

**Figure 4:**
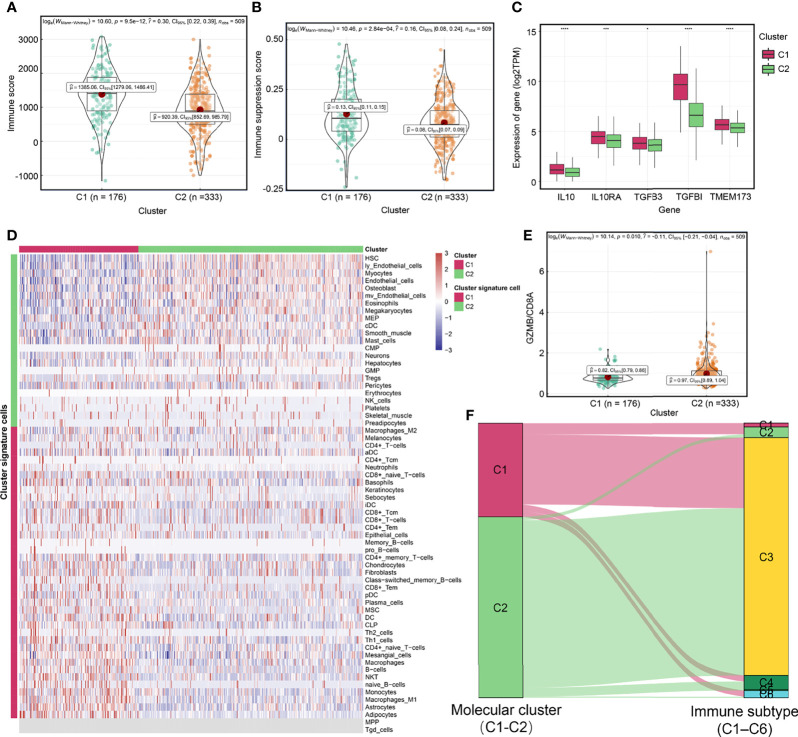
Exploration of extrinsic immune escape mechanisms among molecular clusters. Differences of immune score **(A)** and immune suppression score **(B)** between two molecular clusters. **(C)** Differences of the expression of TMEM173 (STING), IL10, IL10RA, TGFB3 and TGFBI between molecular clusters. **(D)** The relationship between molecular clusters and immune and non-immune cells in the tumor microenvironment. **(E)** The GZMB/CD8A ratio among molecular clusters. **(F)** A Sankey plot was used to reveal the correlation between molecular cluster and immune subtype. (****P < 0.0001; ***P < 0.001; *P < 0.05).

To investigate the mechanisms associated with clinical phenotypic heterogeneity between molecular clusters, we analyzed differences in extrinsic immune escape mechanisms between them. Extrinsic immune escape mechanisms involve four aspects: presence of immunosuppressive cells, lack of immune cells, more fibrosis, and high concentrations of immunosuppressive cytokines, which imply that non-tumor cell components in the TME lead to immune escape of tumor cells (37, 53, 54). **Figures 4A, B** showed that the C1 cluster had higher immune infiltration and immune suppression scores. Expression level of TMEM173 (STING), a signature of spontaneous initiation of innate immunity (37), was higher in the C1 cluster than in the C2 cluster (**Figure 4C**), which were consistent in the E-MTAB-1980 cohort (**Figure S6B** **)**. The C1 cluster exhibited higher infiltrations of immune cells, such as CD8^+^ T-cells, CD4^+^ T-cells, Th1 cells, dendritic cells (DC), and B cells as well as higher infiltrations of immunosuppressive cells, such as Th2 cells, and stromal cells, including fibroblasts and endothelial cells (**Figure 4D** and **Supplement Table 10**). We found that the abundance of Th2 cells of C1 cluster was also higher than that of C2 cluster in the E-MTAB-1980 cohort (**Figure S6C** **)**. Interestingly, the C1 cluster exhibited higher expression levels of chemokines, such as CXCL10, CXCL9 and CCL4, which could attract CD8^+^ T cells and DC (37) (**Supplement Table 11**). Furthermore, immunosuppressive cytokines were upregulated in the C1 cluster, including IL-10 pathway- and TGF-β signaling pathway-related genes (**Figure 4C**), which were basically consistent in the E-MTAB-1980 cohort (**Figure S6B** **)** (35). In contrast, the GZMB/CD8A ratio, a signature of anti-tumor immunity efficacy (55), was significantly higher in the C2 cluster (**Figure 4E**). Thorsson et al. identified six immune subtypes (C1–C6) encompassing 30 cancer types based on the pan-cancer immunogenomics analyses and reveal that the immune subtype C3 was enriched in most ccRCC patients, which characterized by immune equilibrium and a better prognosis than the other immune subtypes (34). Notably, they identified two distinct CD8+ T cell-related molecular clusters, which had distinct proportions of the immune subtype C3, of which C1 cluster-immune subtype C3 accounted for 75.0% and C2 cluster-immune subtype C3 accounted for 92.3% (p<0.001) (**Figure 4F** **)**. Consist with previously reports, our results demonstrated that although less immune cell infiltration, anti-tumor immune components and immune escape components might achieve a balance in the C2 cluster. Overall, although the C1 cluster has a higher immune infiltration level, there are multiple extrinsic immune escape mechanisms that result in worse prognostic outcomes. C2 cluster may represent immune equilibrium and a better prognosis.

### Intrinsic Immune Escape Mechanisms in Molecular Clusters

**Figure 5:**
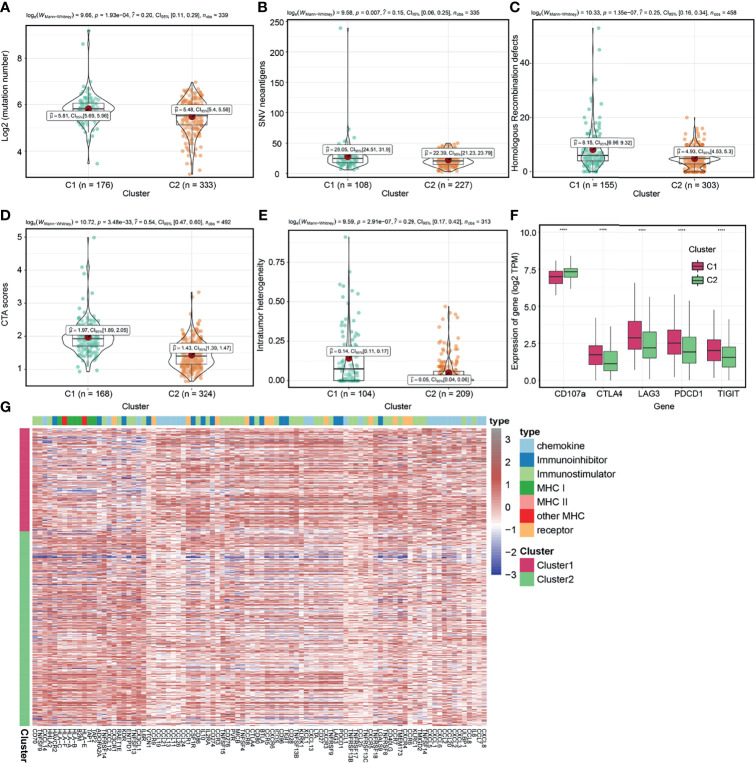
Exploration of intrinsic immune escape mechanisms among molecular clusters. Comparison of tumor mutation burden (TMB) **(A)**, neoantigen load **(B)**, homologous recombination defects (HRD) **(C)**, CTA scores **(D),** and intratumor heterogeneity (ITH) **(E)** among the two clusters. **(F)** Comparison of immunomodulators among the two molecular clusters. **(G)** Heatmap displaying gene clusters of immunomodulators in the two clusters. (****P < 0.0001).

We further evaluated intrinsic immune escape mechanisms between molecular clusters. It has been reported that there are at least two factors that mediate intrinsic immune escape, including immunomodulators and tumor immunogenicity (37, 54). Potential signatures determining tumor immunogenicity were compared between the two clusters: TMB (**Figure 5A**), neoantigen load (**Figure 5B**), HRD (**Figure 5C**), CTA score (**Figure 5D**), ITH (**Figure 5E**), and MHC-related antigen-presenting capability (**Figure 5G**). The C1 cluster had a higher TMB, neoantigen load, HRD, CTA score, and ITH, indicating that it had more sources of tumor antigens. However, when compared to the C2 cluster, the C1 cluster had low expression levels of MHC I- related antigen-presenting genes (**Figure 5G** and **Supplement Table 11**), which contribute to suppressed antigen presentation on tumor surfaces and lower immunogenicity (56, 57). We also found that the expression levels of MHC I- related antigen-presenting genes of C1 cluster was lower than that of C2 cluster in the E-MTAB-1980 cohort (**Figure S6D** **)**. Immunomodulators are involved in other intrinsic immune escape mechanisms and play an essential role in cancer ICB therapy (58). The C1 cluster exhibited elevated expression levels of CD8^+^ T cell exhaustion markers (59), including CTLA4, TIGIT, PDCD1 and LAG3, and suppressed levels of degranulation markers (CD107a) (50) when compared to the C2 cluster (**Figure 5F** and **Supplement Table 11**), which were basically consistent in the E-MTAB-1980 cohort (**Figure S6E** **)**. In addition, we confirmed a closely correlation between the expression levels of most immune checkpoint genes and tumor immunogenicity in ccRCC (**Figure S7**). Overall, the C1 cluster exhibits intrinsic immune escape mechanisms that lead to poor prognostic outcomes.

### Genomic Alterations of Molecular Clusters

**Figure 6:**
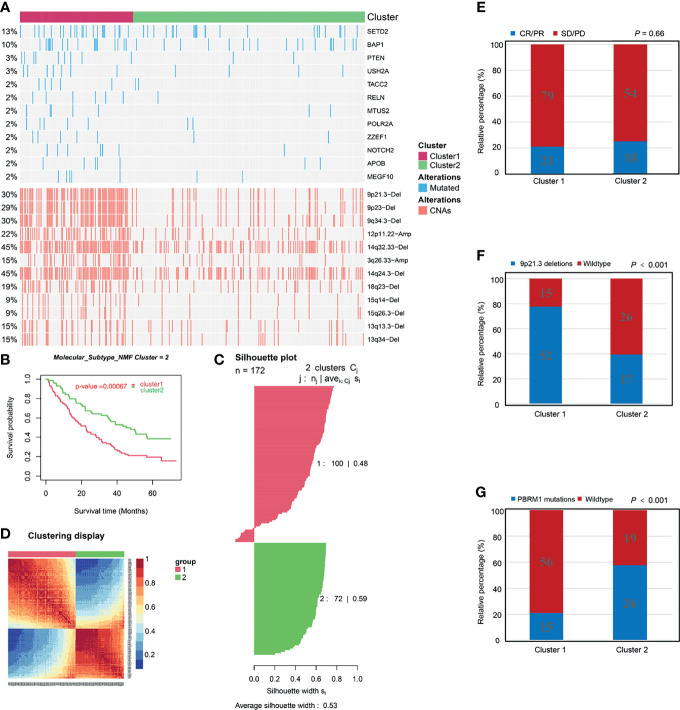
Associations between molecular clusters and immune checkpoint blockade therapy. **(A)** Distribution of driver genes mutation, copy number alterations (CNAs) among the two molecular clusters. **(B-D)** Identification and validation of molecular clusters according to the CD8^+^ T cell-related genes in the Check-mate 025 cohort. **(B)** Kaplan-Meier analysis showed the Cluster1 patients had significantly poorer prognosis than Cluster2 patients. **(C)** Silhouette coefficients indicate that the sample is distinguished from neighboring clusters. **(D)** Differential expression tested the expression difference between two clusters. **(E)** The proportion of patients with response to Nivolumab immunotherapy in C1 and C2 cluster. CR, complete response; PR, partial response; SD, stable disease; PD, progressive disease. **(F)** The proportion of patients with Deletions of 9p21.3 in C1 and C2 cluster. **(G)** The proportion of patients with PBRM1 mutations in C1 and C2 cluster.

Tumoral genomic alterations play a pivotal role in cancer initiation, promotion, progression, and therapy. We assessed differences in genomic alterations between two molecular clusters in the TCGA cohort. First, we compared the NES of 10 oncogenic pathways generated by ssGSEA among the molecular clusters. Cell cycle and TP53- related pathways were highly enriched in the C1 cluster while the Hippo, NRF2, PI3K, RAS and TGF-b- related pathways were enriched in the C2 cluster (**Figure S8A** and **Supplement Table 12**). Subsequently, we identified different frequencies of somatic mutations between the C1 and C2 clusters. Mutations of BAP1, SETD2, TACC2, MTUS2, POLR2A, ZZEF1, APOB, MEGF10, RELN, NOTCH2, USH2A, and PTEN in the C1 cluster were more frequent than in the C2 cluster (**Figure 6A** and **Supplement Table 13**). Loss of PTEN, a tumor suppressor and a member of PI3K-AKT pathway, upregulates the expression of immunosuppressive cytokines and downregulates the expression of IFNG to inhibit T-cell mediated infiltration (56, 60). In addition, PTEN mutation was associated with acquired ICB therapeutic resistance (56, 60). Low expression level of SETD2 is associated with resistance to tyrosine kinase inhibitors (TKIs) in ccRCC patients (61). Our cluster-specific CNAs analysis revealed that chromosomal deletions and amplifications, for example, 9p21.3 deletions were more frequent in the C1 cluster (**Figure 6A** and **Supplement Table 14**). Deletion of 9p21.3 is more enriched in tumors with high immune infiltrations and is associated with worse prognostic outcomes in ccRCC after anti-PD-1 therapy (26), indicating that ICB therapeutic efficacy in the C2 cluster might be higher than in the C1 cluster. Furthermore, we found significantly higher gain and loss of CNAs load in the C1 cluster than in the C2 cluster (**Figure S8B**). High CNAs load has been associated with a more aggressive phenotype of ccRCC (62) and ICB therapeutic resistance (63). Based on these above analyses of genomic alterations, we postulate that poor prognostic outcome of the C1 cluster might be correlated with genomic alterations, and that the C1 cluster might be resistant to ICB therapy.

### Validation of Molecular Classification in the CM-025 Cohort

ICB therapy (e.g., anti-PD-1/PD-L1 therapy) has highly enhanced ccRCC treatment. In the CM-025 cohort the same classification procedure as TCGA and E-MTAB-1980 cohorts was performed to validate molecular classification (**Figures 6B–D**). In addition, IGP values were 84.5% and 82.7% for C1 and C2 clusters, respectively, indicating the high reproducibility of molecular classification in the CM-025 cohort. Kaplan-Meier analysis revealed that the C2 cluster exhibited better OS outcomes (**Figure 6B**) and tended to have higher ICB therapeutic response rates than the C1 cluster (**Figure 6E**), indicating that the C2 cluster is more likely to benefit from anti-PD-1 therapy. The genomic differences between the two clusters in the CM-025 cohort were analyzed (**Figure S9** and **Supplement Tables 15**, **16**). We found that the difference results of 9p21.3 deletion, 9q34.3 deletion and 14q32.33 deletion were consistent with the results of TCGA cohort (**Figure 6F** and **Figure S9**). The VHL and PBRM1 mutations of the C2 cluster were more frequent than those of the C1 cluster (**Figure 6G** and **Figure S9**). It has been proven that the PBRM1 mutations is associated with ICB therapeutic efficacy in ccRCC (26). Overall, we confirm that molecular classification in the CM-025 cohort and the C2 cluster could be used to identify ccRCC patients who are more suitable for anti-PD-1 therapy.

### Construction and Validation of a Gene Classifier

**Figure 7:**
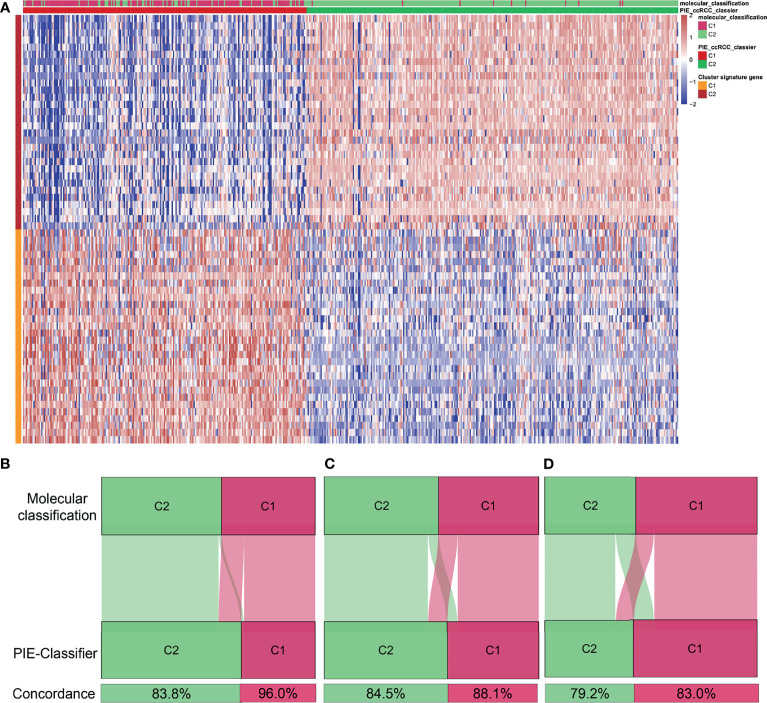
Identification of a simple gene classifier. **(A)** Heatmap of the expression level of the PIE classifier (PIE classifier: prognosis and ICB therapeutic efficacy of the ccRCC classifier). Concordance of molecular clusters prediction in TCGA **(B)**, E-MTAB-1980 **(C)** Check-mate 025 **(D)** cohorts between the PIE classifier and molecular classification based on CNMF algorithm.

To facilitate the clinical application, the NTP algorithm was used to generate the PIE classifier (PIE classifier: prognosis and ICB therapeutic efficacy of the ccRCC classifier) and to predict molecular classification (**Figure 7A** and **Supplement Table 17**) based on the top 30 up-regulated genes in each molecular cluster of TCGA. The PIE classifier was used to predict molecular clusters in TCGA, E-MTAB-1980 and CM-025 cohorts (**Figures 7B–D**). There was a high degree of concordance between prediction results of the PIE classifier and molecular classification, implying that the PIE classifier could simply and reproducibly characterize molecular classification. We also performed Kaplan-Meier analysis of the gene classifier and the results were consistent with the original classification (**Figure S10**).

## Discussion

ccRCC is an immunogenic tumor type which immunologic TME characteristics are relatively unique among solid tumor types (10, 49, 59). There is a high infiltration of CD8^+^ T cells in the TME (22), which are closely associated with the prognosis (59) and ICB therapeutic efficacy of ccRCC (26, 27). However, the infiltrated CD8^+^ T cells exhibit distinct functional status and heterogeneity (27). A lack of patient selection based on CD8^+^ T cell infiltration might be a major reason for low ICB therapeutic efficacy in a considerable proportion of ccRCC patients. In this study, we evaluated the heterogeneous phenotypes of CD8^+^ T cells in ccRCC to enhance the understanding of TME and provide prognostic prediction and ICB therapeutic guidance for ccRCC patients.

Based on CD8^+^ T cell-related genes, two CD8^+^ T cell-related molecular clusters were divided with heterogeneous TME phenotypes and clinical significance. The C1 cluster was characterized by high expression levels of CD8^+^ T cell exhaustion markers, high immune infiltration, and inferior prognosis, as well as more immune escape mechanisms. The C2 cluster was characterized by high expression levels of CD8^+^ T cell effector markers, favorable prognosis, low load of copy number loss, low frequency of 9p21.3 deletion and a high frequency of PBRM1 mutations. Moreover, the C2 cluster exhibited better prognostic outcomes in the CM-025 cohort treated with Nivolumab. Finally, the PIE classifier was generated to predict molecular classification and to facilitate clinical applications. To the best of our knowledge, this study is the first to use multi-omics data to analyze differences in clinical significance and immunogenomic landscape of CD8^+^ T-related molecular patterns.

This study lays the foundation for evaluating potential novel biological functions and mechanisms of CD8^+^ T cells in ccRCC. The mechanisms of blocking the generation of anti-tumor immune responses are quietly complex, theory that have received the most attention is the expression of key receptors on the surface of CD8+ T cells that prevent full CD8+ T cell activation (59). However, few studies have focused on specific role of CD8^+^ T cell related genes in ccRCC immunology. We performed functional enrichment analysis of CD8^+^ T cell related genes and found that CD8^+^ T cells were implicated in multiple biological states or processes, such as chemokine signaling pathway, natural killer cell mediated cytotoxicity, primary immunodeficiency, cytokine-cytokine receptor interaction and hematopoietic cell lineage. Fewer pathways were enriched in the high CD8^+^ T cell- infiltration group than in the low CD8^+^ T cell- infiltration group, implying that more CD8^+^ T cells in the TME of ccRCC were in a state of T cell exhaustion. It has been reported that intratumoral specific CD8^+^ T cell biomarkers can determine the prognosis and immunoevasive outcomes in ccRCC patients, which is a probable explanation for why ccRCC tumors progress despite a robust CD8^+^ T cell infiltration (23, 50, 51). However, the randomly selected markers might not reflect the heterogeneity of CD8^+^ T cells (50, 51, 64). In this study, WGCNA was used to identify CD8^+^ T cell related module genes, while the unsupervised clustering method was used to better identify novel CD8^+^ T cell-related molecular patterns. The TMB, neoantigen load and immune checkpoint gene expression might not be significantly associated with ICB therapeutic efficacy for ccRCC (10, 49), but were closely associated with CD8^+^T cells (27, 50, 51). Notably, according to the association between CD8^+^ T cells and TMB, neoantigen load and immune checkpoint gene expression, we found that the CD8^+^ T cells and checkpoint molecule expression, but not mutation or neoantigen loads, might be better predictors for ICB therapy. The high CD8^+^ T cell- infiltration group exhibited lower expression level of CD207a and higher expression levels of critical immune checkpoint genes, immune suppression scores and an abundance of immunosuppressive cells (M2 macrophages and Th2 cells), which may explain the poor prognosis of ccRCC with high CD8^+^ T cell infiltration (22, 23). Previous studies have also proved that high infiltration levels of CD8^+^ T cells in ccRCC were associated with elevated expression levels of immune evasive biomarkers and enhanced immunosuppressive cell infiltrations (24, 25). Overall, infiltrating CD8^+^ T cells in ccRCC exhibit an immunosuppressed phenotype, which is a probable explanation for why ccRCC tumors progress despite robust T cell infiltrations.

Our study has practical clinical implications for prognostic prediction of ccRCC patients. We used the CNMF algorithm, based on CD8^+^ T cell-related genes, to identify two distinct molecular clusters. The OS outcomes of the C2 cluster were significantly better than those of the C1 cluster. Furthermore, molecular classification was shown to better predict OS outcomes for ccRCC. Previous studies identified ccRCC clusters based on genomic profiling (32, 65, 66), thereby improving the ability of clinicians to make personalized treatment decisions. Samira et al. constructed a ClearCode34 classifier to stratify ccRCC patients into good risk (ccA) and poor risk (ccB) subtypes (32). Compared to their classifications, our molecular classification exhibited higher AUC and C-index, but were not significantly different. Moreover, the DCA for 5-year OS prediction revealed that the net benefit of molecular classification was higher than that of ClearCode34. Finally, we validated molecular classification in the E-MTAB-1980 cohort, and found that the two molecular clusters had a high degree of consistency between the two cohorts.

To evaluate the mechanisms that contribute to different prognostic phenotypes of CD8^+^ T cell-related molecular clusters, we performed immunogenomic landscape analyses. The C1 cluster exhibited elevated immune infiltrations and more extrinsic and intrinsic immune escape mechanisms than the C2 cluster. Heterogeneities of the TME phenotypes led to different clusters of ccRCC, with distinct tumor immune escape mechanisms. Based on the immunoediting theory, it has been shown that a lack of immune cells, presence of immune-inhibitory cells, high concentrations of immune-inhibitory cytokines, and fibrosis might be attributed to extrinsic immune escape mechanisms of tumors (37, 53, 54). The C1 cluster exhibited not only more infiltrations of anti-tumor immune cells, but also more infiltrations of immunosuppressive cells and stromal cells including fibroblasts and endothelial cells. From the perspective of prognosis, it was speculated that immunosuppressive cells were dominant in C1 cluster, leading to a worse prognosis. In the C2, although less immune cell infiltration, but the dominant position in the anti-tumor immune cells, which may account for the better prognosis. Based on comparisons with immune subtypes proposed by Thorsson et al. (34), we further speculate that the C2 cluster may represent immune equilibrium. In addition, we also analyzed deeply differences of the other TME signatures (e.g metabolic reprograming, immunogenicity related signatures, somatic mutation and copy number variation) between the two clusters, and revealed that a deeply relationship between them. However, the specific mechanisms involved in alterations of these microenvironment components should be further investigated.

Tumor cells adapt their metabolism to support increased bioenergetic needs and biosynthesis requirements of proliferation and invasiveness. Metabolic reprograming is a pivotal mechanism for immune escape in several human malignancies, resulting in poor prognosis and low ICB therapeutic responses (67–69). Metabolic alterations in the TME lead to competition for nutrients and oxygen of immune and cancer cells. Furthermore, cancer cells and surrounding cells infiltrated in the tumor secrete metabolites and inhibitory cytokines that interfere with the targeting of immune cells to eliminate tumor cells. Ultimately, tumor cells create a favorable environment for their growth and development while evading and suppressing the immune response (70). For example, high levels of serum metabolite (e.g hypoxanthine and histidine) were associated with improved progression-free survival and may serve as predictive biomarkers of response to PD-1 blockade therapy in advanced NSCLC patients (71). Lipid accumulation was correlated with elevated expression of CD36, a scavenger receptor for oxidized lipids, on CD8^+^ TILs, which also correlated with progressive T cell dysfunction (72). Consistent with these reports, the GESA showed that both of two CD8^+^ T cell-based molecular clusters were correlated with metabolism related pathways. The C1 cluster type was enriched in immunosuppressive hallmarks including JAK/STAT3 signaling, Toll like receptor signaling, VEGF signaling, hedgehog signaling and p53 pathways. These results suggest that the C1 cluster might develop cancer immune escape related to immunosuppression in TME and multiple malignancy hallmarks, resulting in adverse clinical outcomes and poor immune responses. The C2 cluster was correlated with amino acid metabolism which would enable early identification of ccRCC patients who may benefit from PD-1 blockade therapy. Based on the above results, we postulate that the enriched lipids metabolism promotes intratumoral CD8^+^ T cell dysfunction and may serve as a therapeutic avenue for immunotherapies.

This study may contribute to the selection of ccRCC patients who are suitable for immunotherapy. Based on comparisons with immune subtypes proposed by Thorsson et al. (34), we further speculate that the C2 cluster may represent immune equilibrium. The C2 cluster exhibited a lower immunogenicity and elevated MHC I- related antigen-presenting gene expression than the C1 cluster, implying that the C2 cluster is suitable for immunotherapy (57). Through the analysis of genomic alterations of molecular clusters, we further found that the C2 cluster was more suitable for immunotherapy. The C2 cluster was characterized by a lower load of copy number loss and low frequencies of 9p21.3 deletion in the TCGA cohort, which might be correlated with improved ICB therapeutic efficacy (26, 63). In addition, the C2 cluster of the CM-025 cohort exhibited a low frequency of 9p21.3 deletions while PBRM1 mutations in the C2 cluster were more frequent than those of the C1 cluster. Moreover, the C2 cluster exhibited favorable OS outcomes in the CM-025 cohort. A series of biomarkers, including PD1/PD-L1 expression, TMB, and microsatellite instability (MSI) are potential prognostic indicators for solid tumor ICB therapeutic outcomes (73, 74). However, there was no clear correlation between these markers and the efficacy of immunotherapy for ccRCC (10, 73). We found that molecular classification could provide ICB therapeutic guidance for ccRCC patients as well as a basis for clinical trials. To facilitate clinical applications, we established a simple gene classifier, the PIE classifier, to predict molecular classification. The gene classifier needs to be further optimized to increase or decrease the number of up-regulated genes of clusters, so as to achieve more accurate prediction of molecular classification.

There are some limitations in our research. First, in the retrospective cohort, the sample size was not large enough, although more cohorts were included for validation. In addition, CD8^+^ T cell abundance was estimated based on bulk sequencing, and CD8^+^ T cell-related genes were not verified by single cell RNA sequencing. Immunogenomic analysis between molecular clusters could not directly reflect causality, which should be verified by relevant experiments.

In summary, we evaluate the potential biological functions of CD8^+^ T cells in ccRCC to enhance our understanding of TME. The TME phenotypes of ccRCC could be classified into two CD8^+^ T-cell related molecular clusters with heterogeneous immunogenomic landscapes and clinical significance. Molecular classification can be used for prognostic prediction and ICB therapeutic guidance of ccRCC patients.

## Data Availability Statement

The datasets presented in this study can be found in online repositories. The names of the repository/repositories and accession number(s) can be found in the article/**Supplementary Material**.

## Author Contributions

LL designed the study. XW, DL, and HL contributed to data analysis and interpretation. DJ and XL assisted in collection and assembly of data. XW and DL wrote and edited the manuscript. LL and DL obtained funding support. All authors participated in preparing the manuscript and approved the final manuscript.

## Funding

This study was funded by grants from the National Key R&D program of China (Grant NO. 2017YFC1309002), the Guangdong Basic and Applied Basic Research Foundation (Grant NO. 2019A1515110033), China Postdoctoral Science Foundation (Grant NO. 2019M662865), Distinguished Young Talents in Higher Education Foundation of Guangdong Province (Grant NO. 2019KQNCX115), Science and Technology Plan Project of Guangzhou (Grant NO. 202102010150 and 202102080010), Achievement cultivation and clinical transformation application cultivation projects of the First Affiliated Hospital of Guangzhou Medical University (Grant NO. ZH201908).

## Conflict of Interest

The authors declare that the research was conducted in the absence of any commercial or financial relationships that could be construed as a potential conflict of interest.

## Publisher’s Note

All claims expressed in this article are solely those of the authors and do not necessarily represent those of their affiliated organizations, or those of the publisher, the editors and the reviewers. Any product that may be evaluated in this article, or claim that may be made by its manufacturer, is not guaranteed or endorsed by the publisher.

## Supplementary Material

Supplementary Figure 1
**(A)** The flowchart of this study. **(B)** The volcano plot was used to visualize the immune-related differentially expressed genes (IRDEGs) between ccRCC and normal tissue samples in TCGA. **(C)** Intersection of immune-related genes and IRDEGs. (TCGA, The Cancer Genome Atlas).

Supplementary Figure 2 Kaplan-Meier analysis of 20 types of immune and non-immune cells.

Supplementary Figure 3
**(A–G)** Correlation between abundance of CD8^+^ T cells and immune-related signatures. **(H)** The relationship between tumor mutation burden (TMB) and abundance of CD8^+^ T cells. **(I)** The relationship between neoantigen load and abundance of CD8^+^ T cells.

Supplementary Figure 4
**(A–C)** Silhouette coefficients indicate that the sample is distinguished from neighboring clusters in TCGA cohort. **(D)** The relationship between abundance of CD8^+^ T cells and expression of 84 prognostic genes. The comparison of area under the curve (AUC) **(E)** and concordance index (C-index) **(F)** between molecular cluster and ClearCode34 classification.

Supplementary Figure 5 Identification and validation of molecular clusters according to the CD8^+^ T cell-related genes in the E-MTAB-1980 cohort. **(A)**. Kaplan-Meier analysis showed the Cluster1 patients had significantly poorer prognosis than Cluster2 patients. **(B)**. Silhouette coefficients near 1 indicate that the sample is distinguished from neighboring clusters and determine that the best number of clusters was two. **(C)**. Differential expression tested the expression difference between two clusters. **(D–F)** Silhouette coefficients indicate that the sample is distinguished from neighboring clusters.

Supplementary Figure 6
**(A)**. Gene set variation analysis (GSVA) between C1 cluster and C2 cluster in TCGA cohort. **(B)** Differences of the expression of TMEM173 (STING), IL10, IL10RA, TGFB3 and TGFBI between molecular clusters in E-MTAB-1980 cohort. **(C)** Differences of abundance of Th2 cells between molecular clusters in TCGA cohort. **(D)** The expression levels of MHC I- related antigen-presenting genes of C1 cluster was lower than that of C2 cluster in the E-MTAB-1980 cohort. **(E)** Differences of the expression of CD107a, CTLA4, LAG3, PDCD1 and TIGIT between molecular clusters in E-MTAB-1980 cohort. (****, *P* < 0.0001; ***, *P* < 0.001; **, *P* < 0.01; *, *P* < 0.05).

Supplementary Figure 7 Correlations between expression levels of immune checkpoint genes and immunogenicity.

Supplementary Figure 8 Differences of ssGSEA score of 10 oncogenic pathways among molecular clusters in TCGA cohort. **(B)** Comparison of gain and loss of copy-number alterations load among two molecular clusters. (****, P < 0.0001; ***, P < 0.001; **, P < 0.01; *, P < 0.05) (ssGSEA: Single sample Gene Set Enrichment analysis).

Supplementary Figure 9 Distribution of copy number alterations (CNAs) **(A)** and driver genes mutation **(B)** among the two molecular clusters in the CM-025 cohort.

Supplementary Figure 10 Kaplan-Meier analysis of the PIE classifier of TCGA **(A)**, E-MTAB-1980 **(B)** and CM-025 **(C)** cohorts. (PIE classifier: prognosis and ICB therapeutic efficacy of the ccRCC classifier).

## Abbreviations

ccRCC, Clear cell renal cell carcinoma; ICB, immune checkpoint blockade; TCGA, The Cancer Genome Atlas; WGCNA, Weighted gene co-expression network analysis; ssGSEA, Single sample Gene Set Enrichment analysis; GSVA, Gene set variation analysis.
