# Supplementary figures and images for: CD8+ T Cell-Based Molecular Classification With Heterogeneous Immunogenomic Landscapes and Clinical Significance of Clear Cell Renal Cell Carcinoma
Source: Front Immunol. 2021 Dec 14;12:745945. doi: 10.3389/fimmu.2021.745945 (PMC8713701; doi:10.3389/fimmu.2021.745945)

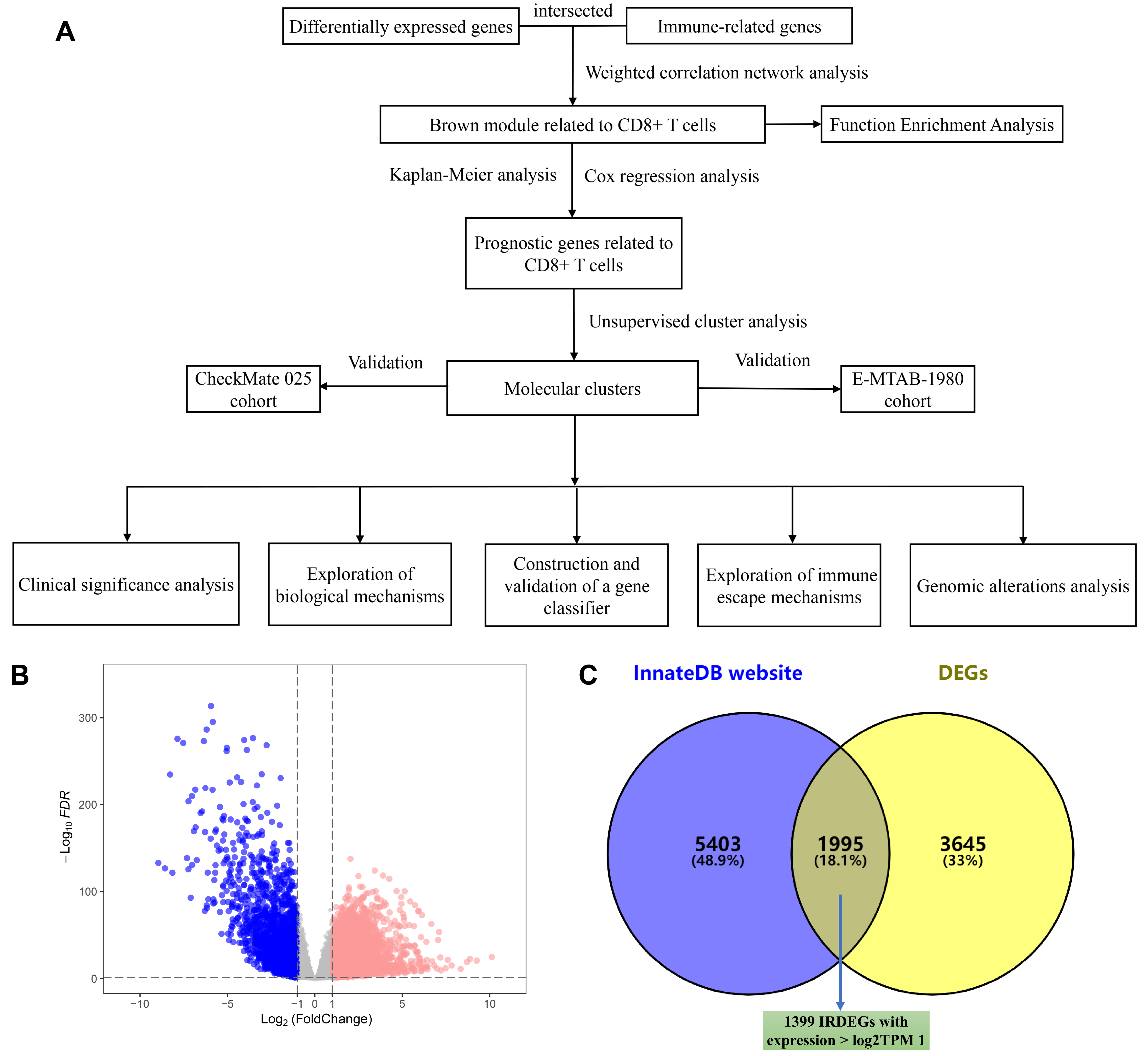

Supplement: Supplementary Figure 1 — (A) The flowchart of this study. (B) The volcano plot was used to visualize the immune-related differentially expressed genes (IRDEGs) between ccRCC and normal tissue samples in TCGA. (C) Intersection of immune-related genes and IRDEGs. (TCGA, The Cancer Genome Atlas). [file Image_1.tif]

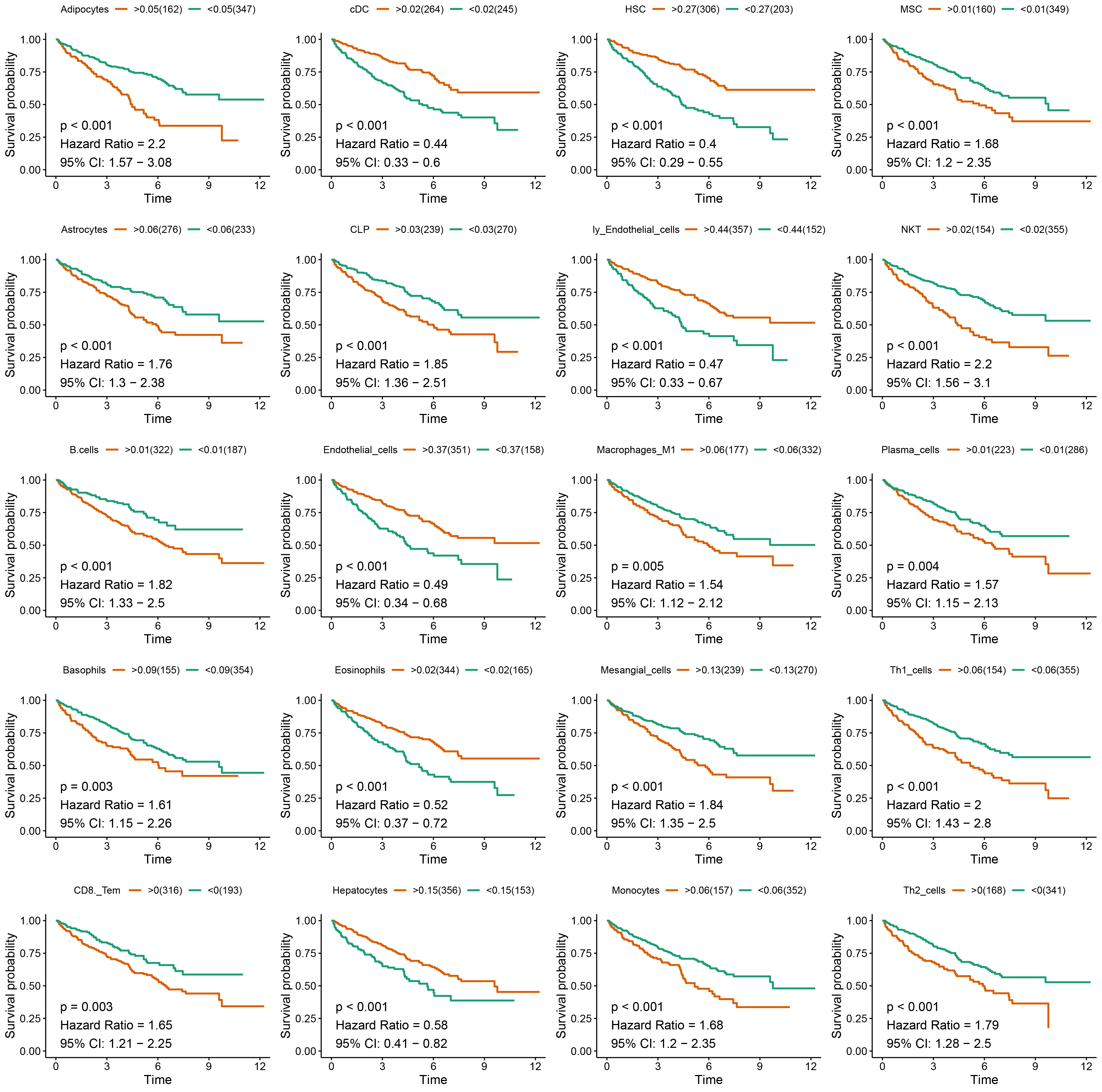

Supplement: Supplementary Figure 2 — Kaplan-Meier analysis of 20 types of immune and non-immune cells. [file Image_2.tif]

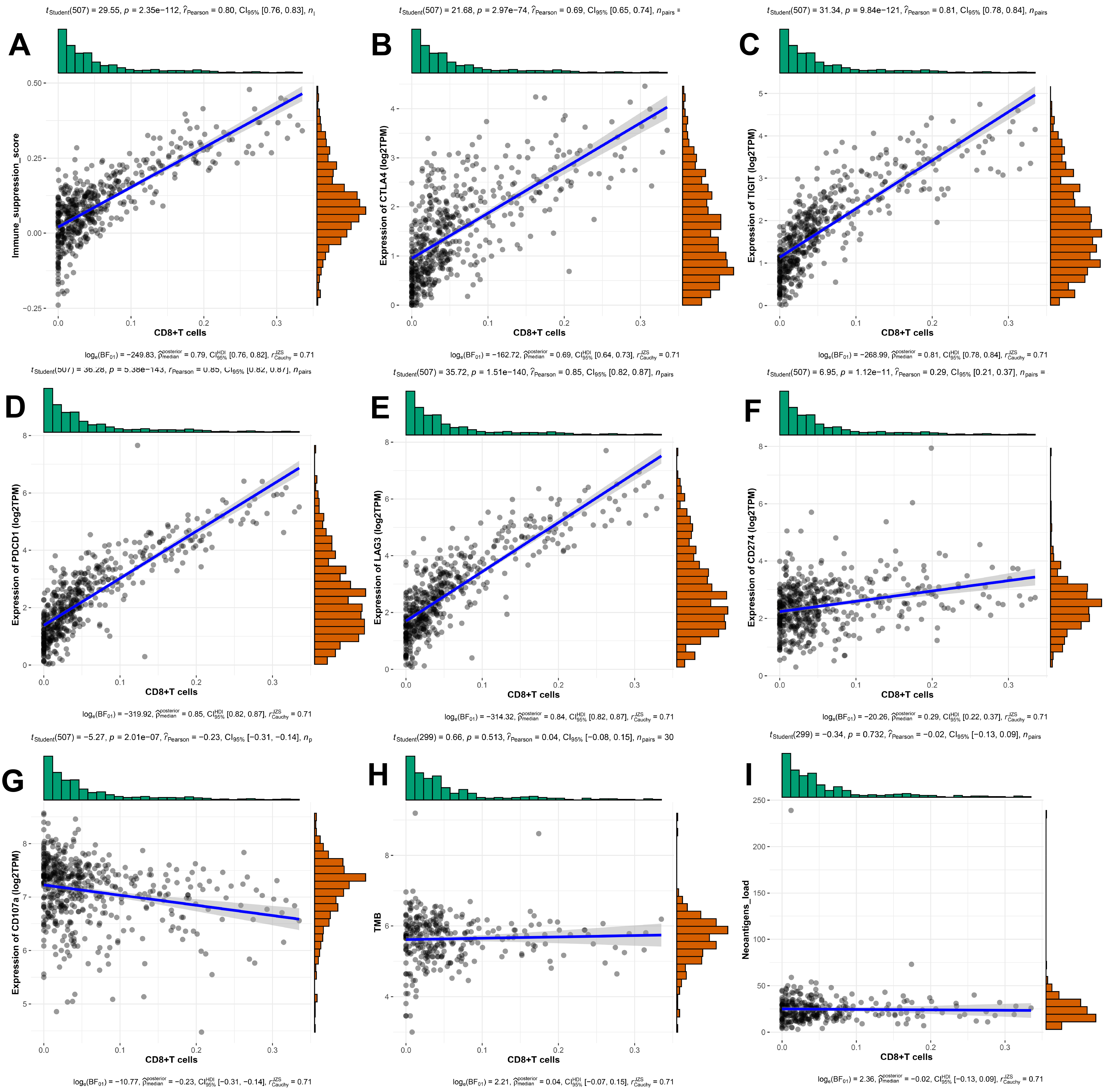

Supplement: Supplementary Figure 3 — (A–G) Correlation between abundance of CD8+ T cells and immune-related signatures. (H) The relationship between tumor mutation burden (TMB) and abundance of CD8+ T cells. (I) The relationship between neoantigen load and abundance of CD8+ T cells. [file Image_3.tif]

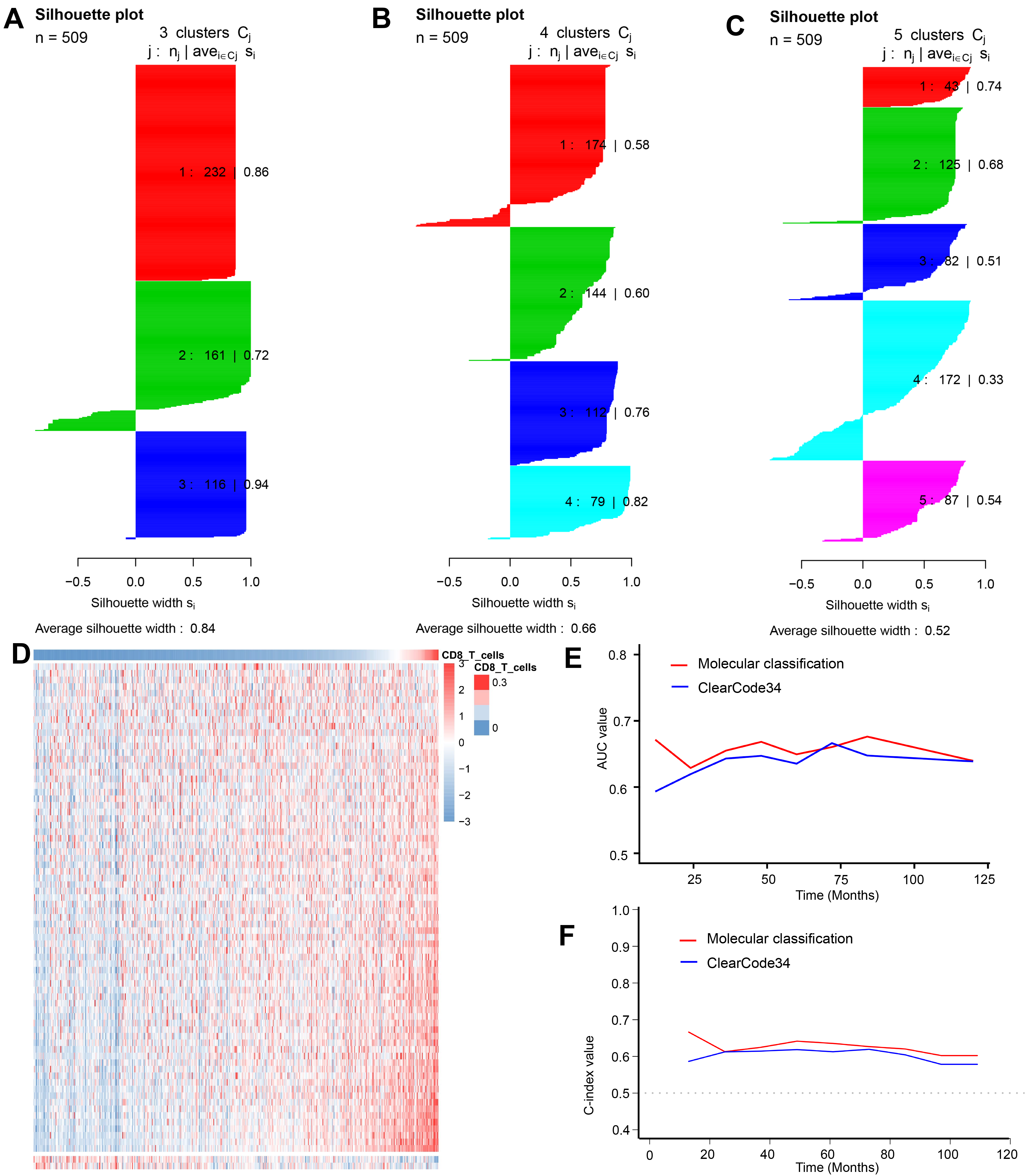

Supplement: Supplementary Figure 4 — (A–C) Silhouette coefficients indicate that the sample is distinguished from neighboring clusters in TCGA cohort. (D) The relationship between abundance of CD8+ T cells and expression of 84 prognostic genes. The comparison of area under the curve (AUC) (E) and concordance index (C-index) (F) between molecular cluster and ClearCode34 classification. [file Image_4.tif]

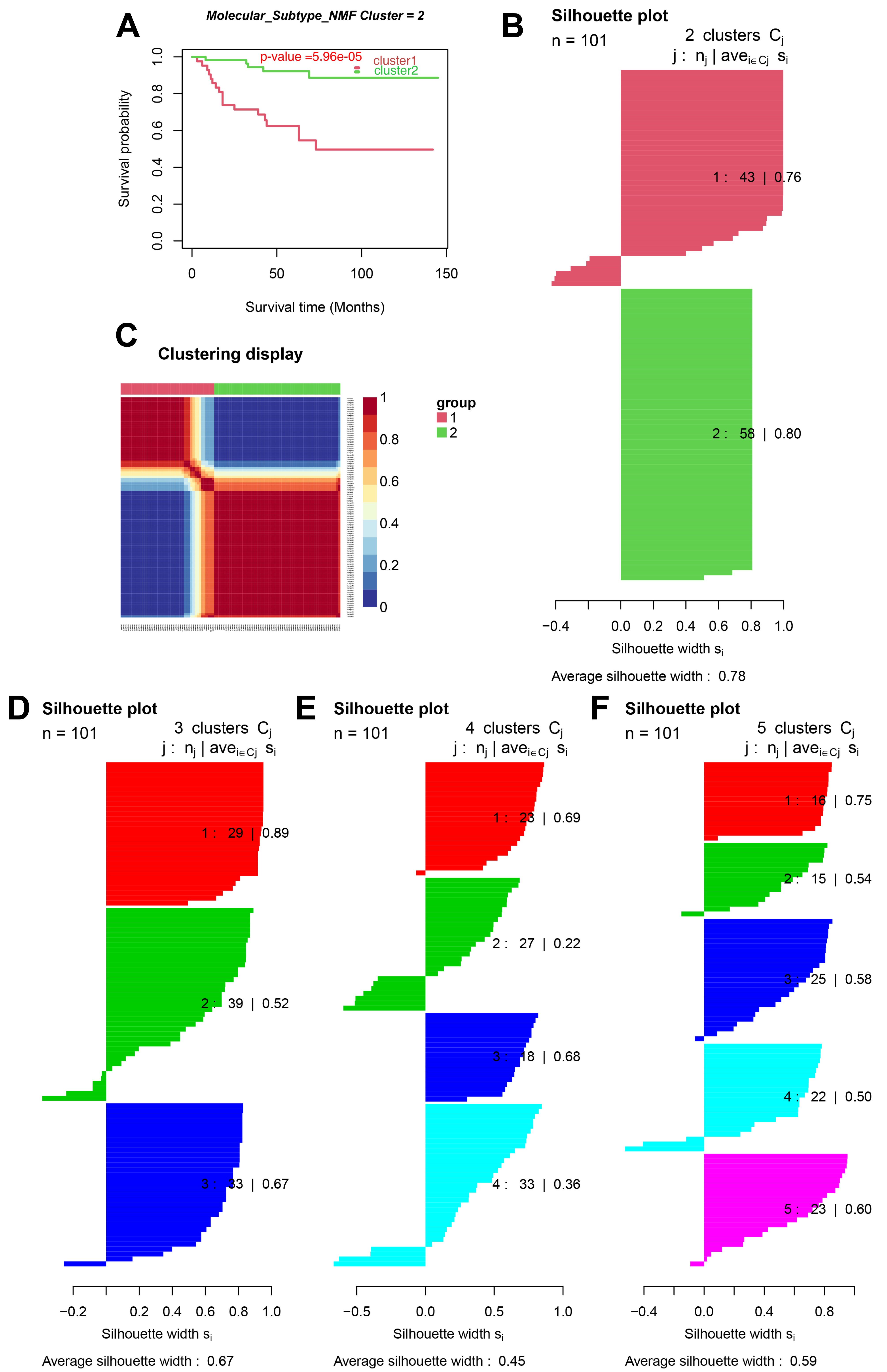

Supplement: Supplementary Figure 5 — Identification and validation of molecular clusters according to the CD8+ T cell-related genes in the E-MTAB-1980 cohort. (A). Kaplan-Meier analysis showed the Cluster1 patients had significantly poorer prognosis than Cluster2 patients. (B). Silhouette coefficients near 1 indicate that the sample is distinguished from neighboring clusters and determine that the best number of clusters was two. (C). Differential expression tested the expression difference between two clusters. (D–F) Silhouette coefficients indicate that the sample is distinguished from neighboring clusters. [file Image_5.tif]

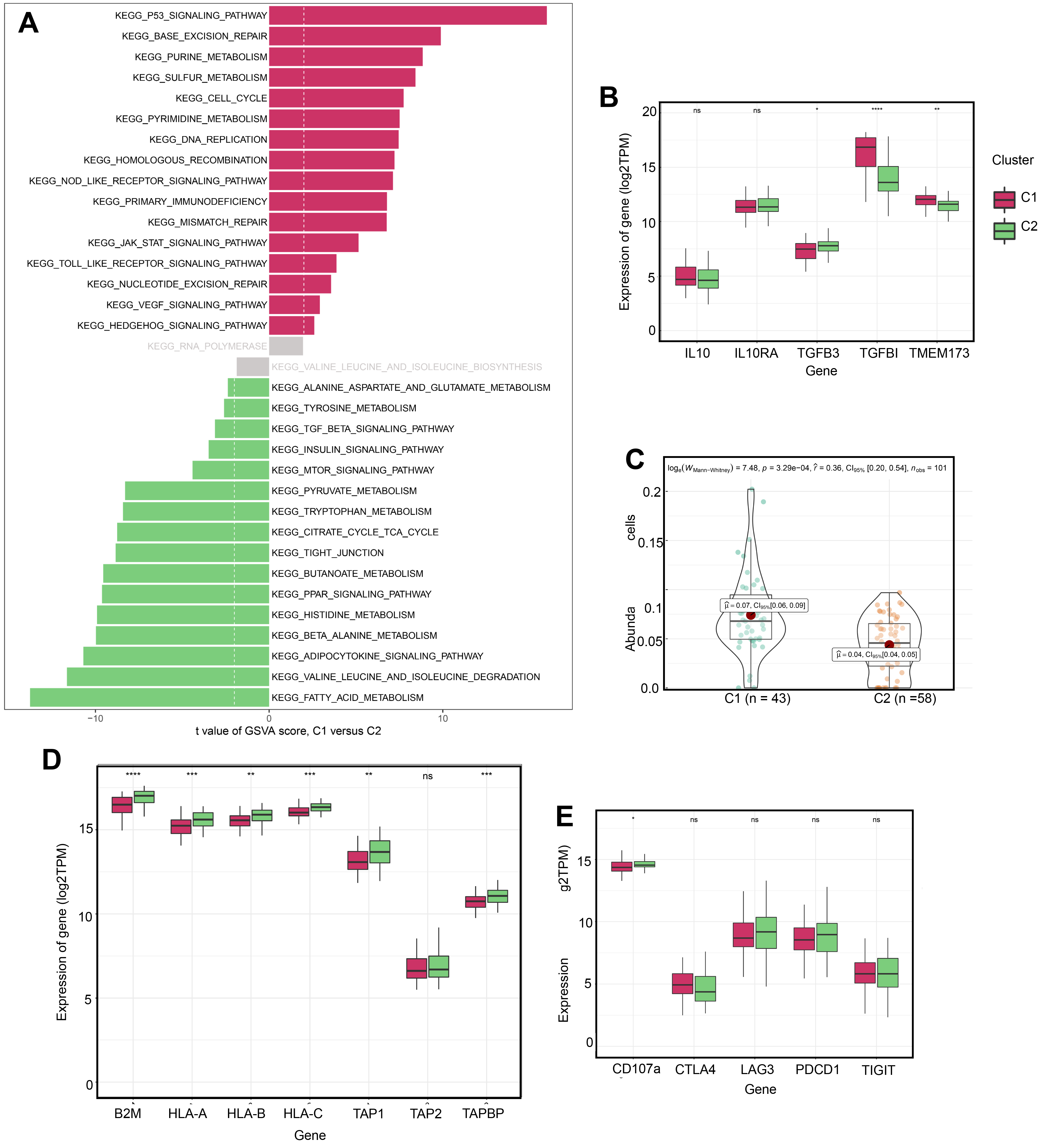

Supplement: Supplementary Figure 6 — (A). Gene set variation analysis (GSVA) between C1 cluster and C2 cluster in TCGA cohort. (B) Differences of the expression of TMEM173 (STING), IL10, IL10RA, TGFB3 and TGFBI between molecular clusters in E-MTAB-1980 cohort. (C) Differences of abundance of Th2 cells between molecular clusters in TCGA cohort. (D) The expression levels of MHC I- related antigen-presenting genes of C1 cluster was lower than that of C2 cluster in the E-MTAB-1980 cohort. (E) Differences of the expression of CD107a, CTLA4, LAG3, PDCD1 and TIGIT between molecular clusters in E-MTAB-1980 cohort. (****, P < 0.0001; ***, P < 0.001; **, P < 0.01; *, P < 0.05). [file Image_6.tif]

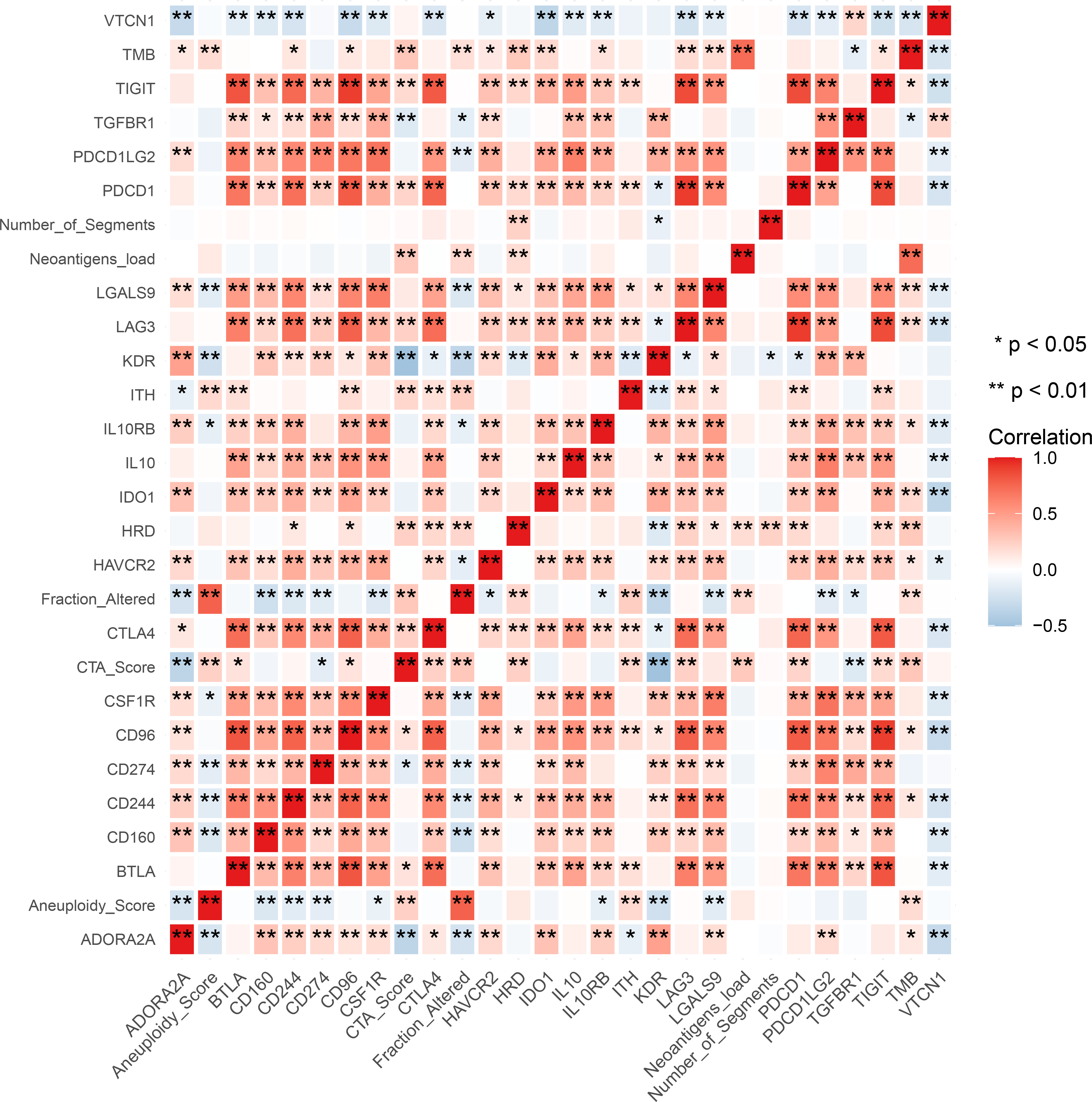

Supplement: Supplementary Figure 7 — Correlations between expression levels of immune checkpoint genes and immunogenicity. [file Image_7.tif]

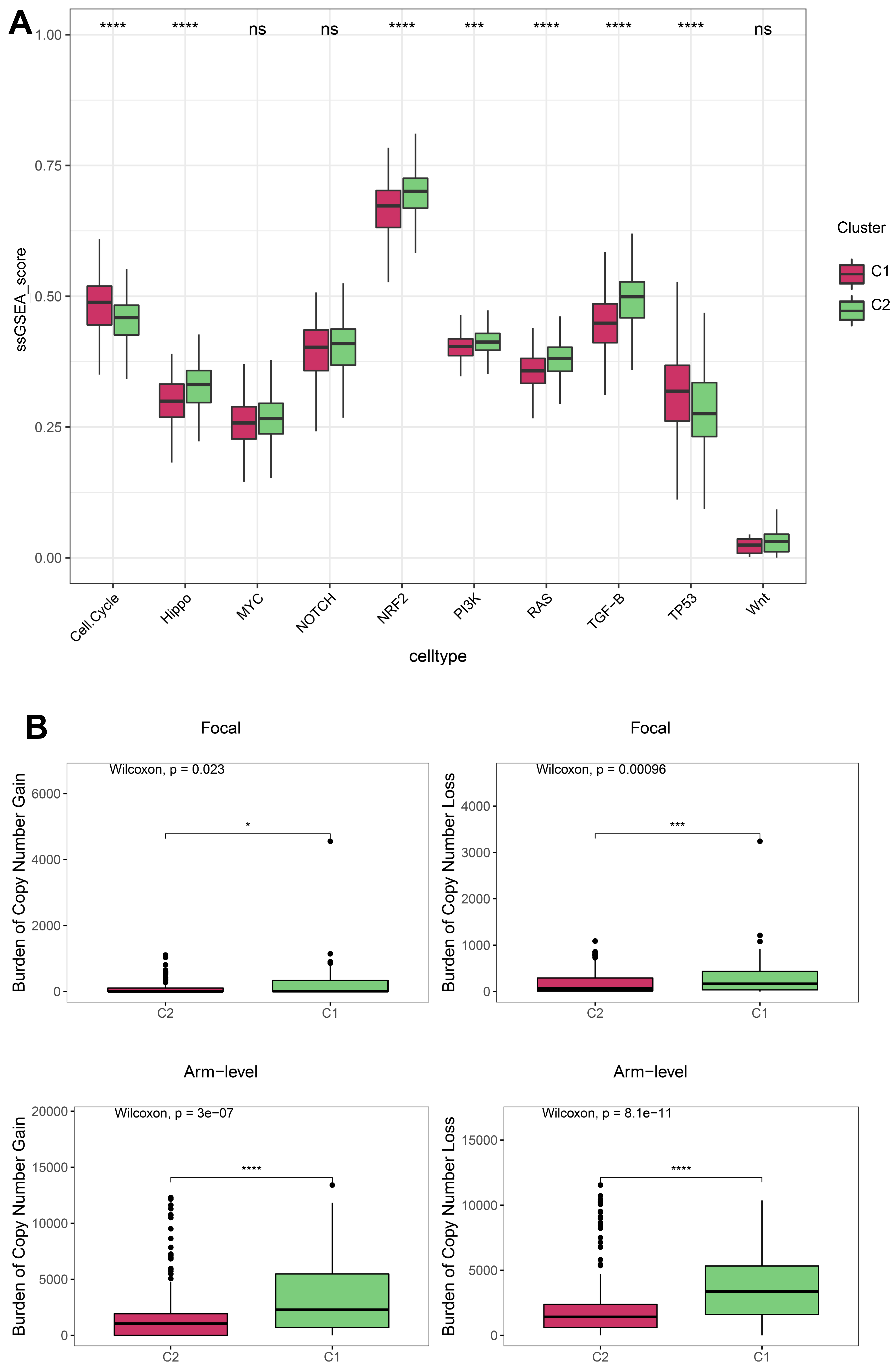

Supplement: Supplementary Figure 8 — Differences of ssGSEA score of 10 oncogenic pathways among molecular clusters in TCGA cohort. (B) Comparison of gain and loss of copy-number alterations load among two molecular clusters. (****, P < 0.0001; ***, P < 0.001; **, P < 0.01; *, P < 0.05) (ssGSEA: Single sample Gene Set Enrichment analysis). [file Image_8.tif]

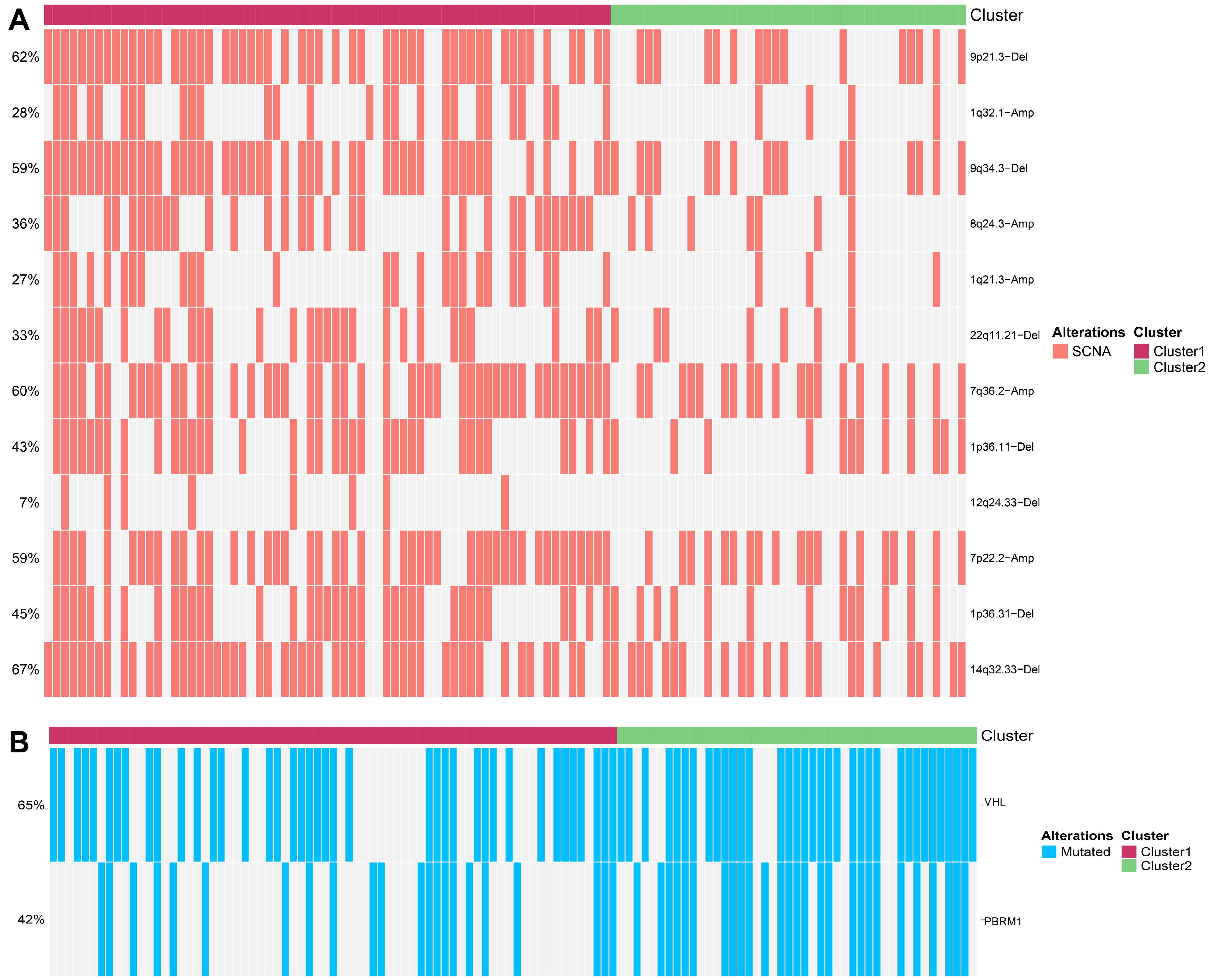

Supplement: Supplementary Figure 9 — Distribution of copy number alterations (CNAs) (A) and driver genes mutation (B) among the two molecular clusters in the CM-025 cohort. [file Image_9.tif]

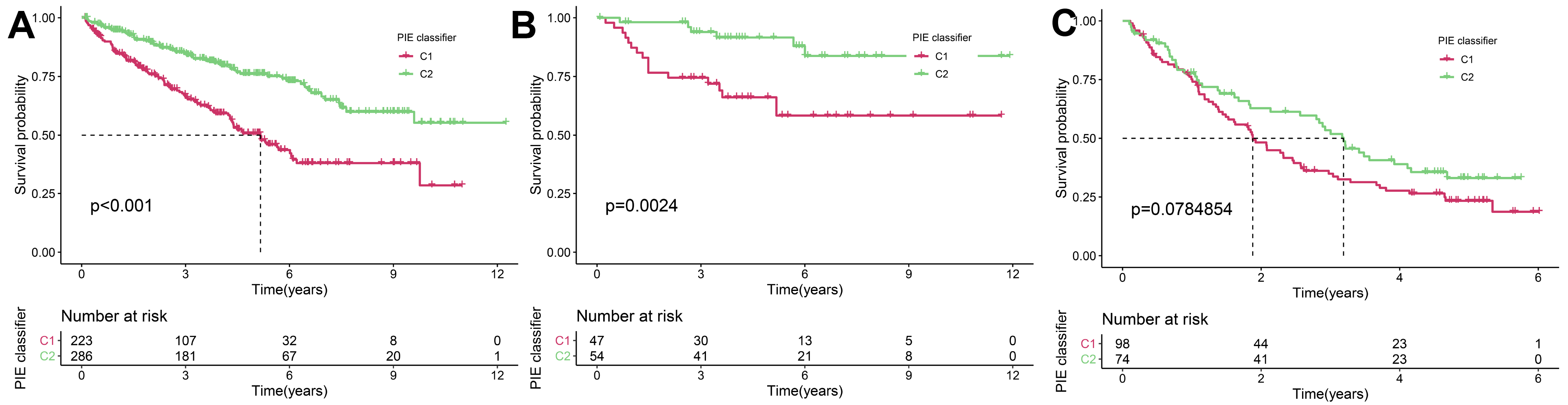

Supplement: Supplementary Figure 10 — Kaplan-Meier analysis of the PIE classifier of TCGA (A), E-MTAB-1980 (B) and CM-025 (C) cohorts. (PIE classifier: prognosis and ICB therapeutic efficacy of the ccRCC classifier). [file Image_10.tif]
